# Organotypic Epithelial Raft Cultures as a Three-Dimensional In Vitro Model of Merkel Cell Carcinoma

**DOI:** 10.3390/cancers14041091

**Published:** 2022-02-21

**Authors:** Arturo Temblador, Dimitrios Topalis, Joost van den Oord, Graciela Andrei, Robert Snoeck

**Affiliations:** 1 Laboratory of Virology and Chemotherapy, Department of Microbiology, Immunology and Transplantation, Rega Institute for Medical Research, KU Leuven, 3000 Leuven, Belgium; artu15arcos@hotmail.com (A.T.); dimitri.topalis@gmail.com (D.T.); robert.snoeck@kuleuven.be (R.S.); 2 Laboratory of Translational Cell and Tissue Research, Department of Imaging and Pathology, KU Leuven, 3000 Leuven, Belgium; joost.vandenoord@kuleuven.be

**Keywords:** non-melanoma skin cancer, Merkel cell carcinoma, Merkel cell polyomavirus, 3D cell culture model, raft culture, gene expression profile

## Abstract

**Simple Summary:**

Merkel cell carcinoma (MCC) is an extremely aggressive type of skin cancer. Nevertheless, the development of new therapeutic approaches to treat this malignancy is hampered by the absence of in vitro culture models. Furthermore, prospective clinical trials are scarce due to the rarity of the disease. Thus, the aim of the present study was to generate organotypic epithelial raft cultures (OERCs) of MCC by growing primary human keratinocytes and MCC cell lines on artificial dermal equivalents. Histological and immunohistochemical analyses confirmed the proliferation of MCC cell lines. In addition, gene expression analysis showed genes that were differently expressed in the tumor cells and the keratinocytes. In brief, we developed a three-dimensional cell culture model of MCC that can potentially be used for evaluating the efficacy and selectivity of new drug candidates against this disease.

**Abstract:**

Merkel cell carcinoma (MCC) is a rare type of skin cancer for which an in vitro model is still lacking. MCC tumorigenesis is associated either with the integration of Merkel cell polyomavirus into the host genome, or with the accumulation of somatic mutations upon chronic exposure to UV light. Transgenic animals expressing the viral oncoproteins, which are constitutively expressed in virus-related MCC, do not fully recapitulate MCC. Although cell-line-derived xenografts have been established for the two subtypes of MCC, they still present certain limitations. Here, we generated organotypic epithelial raft cultures (OERCs) of MCC by using primary human keratinocytes and both virus-positive and virus-negative MCC cell lines. The primary human keratinocytes and the tumor cells were grown on top of a dermal equivalent. Histological and immunohistochemical examination of the rafts confirmed the growth of MCC cells. Furthermore, gene expression analysis revealed differences in the expression profiles of the distinct tumor cells and the keratinocytes at the transcriptional level. In summary, considering the limited availability of patient samples, OERCs of MCC may constitute a suitable model for evaluating the efficacy and selectivity of new drug candidates against MCC; moreover, they are a potential tool to study the oncogenic mechanisms of this malignancy.

## 1. Introduction

Merkel cell carcinoma (MCC) is an aggressive type of skin tumor with a mortality rate between 33 and 46% [1]. MCC’s incidence is higher among elderly individuals of Caucasian origin with prolonged exposure to UV light. Despite MCC’s incidence being rather low (i.e., 0.7 cases/100,000 individuals in the US), several studies have reported increasing incidence, which is projected to persist in the coming years [2].

Little was known about MCC tumorigenesis until the identification of a new human polyomavirus in 2008—Merkel cell polyomavirus (MCPyV), clonally integrated in the majority of the tumors [3]. MCPyV-positive (MCPyV^+^) MCC constitutively expresses two viral oncoproteins: the small (sT) and the large (LT) tumor antigens (TAs). Similar to other polyomaviruses capable of inducing tumors in animal models (e.g., SV40) [4], a truncated form of the LT of MCPyV, which conserves the LXCXE motif, maintains cell growth by inhibiting retinoblastoma (Rb) activity [5,6]. Moreover, the sT also exerts several functions contributing to tumorigenesis [1]. MCPyV-negative (MCPyV^−^) MCC is predominant in Australia and New Zealand [1,7], supposedly due to the role of sun irradiation in the accumulation of the somatic mutations that give rise to this subgroup [8,9,10].

The current models used to study MCC biology and treatment are limited to monolayer cell cultures and xenografts in immunodeficient mice [11,12]. MCC cell-line-derived xenograft (CDX) mouse models have been developed for both MCPyV^+^ and MCPyV^−^ MCC cell lines. Generally, MCC cells are suspended in Matrigel and then subcutaneously injected in NOD/SCIDγ (NSG) mice to induce tumor formation. This model has proved useful for testing novel drugs against MCC [11,13,14,15,16]. MCC patient-derived xenograft (PDX) mouse models contain subcutaneously implanted MCC tissue. Since the tissue is derived from a patient with MCC, one major advantage of this model is the conservation of many characteristics of the tumor of origin. Thus, PDXs are a powerful tool to predict a clinical response to a chosen drug. In this regard, copanlisib—an inhibitor of PI3K-α and -δ isoforms that is FDA-approved for refractory follicular lymphoma—has shown antitumor activity in MCC PDXs [17].

The heterogeneity of the mutations that lead to MCPyV^−^ MCC hampers the development of animal models for this malignancy. Regarding the virus-related counterpart, several studies have attempted to recapitulate MCPyV^+^ MCC tumorigenesis in vivo by expressing the viral oncoproteins in genetically engineered mice. Strikingly, only MCPyV sT showed transforming activity in rat-1 fibroblasts and mouse models [8,18,19,20]. Moreover, co-expression of MCPyV sT with the transcription factor Atoh1 induced the development of lesions resembling MCC in mice, without the need for LT expression. Nevertheless, progress is needed to develop a mouse model closely reflecting the human MCC [21].

While animal models are time-consuming, expensive, and bear ethical considerations, three-dimensional (3-D) in vitro cell culture models represent a feasible alternative. Recently, three MCPyV^+^ MCC cell lines (MKL-1, WAGA, and PeTa) have been successfully grown in a 3D cell culture model—the avian chorioallantoic membrane (CAM). The CAM represents a rich vascular network that can vascularize the tumor without executing an immune response against the graft; thus, this model allows the investigation of MCC angiogenesis and the tumor microenvironment [22,23,24].

Organotypic epithelial raft cultures (OERCs) are 3D in vitro culture systems that permit terminal differentiation of keratinocytes—the major cell type of the epidermis—when seeded on top of collagen beds at the air–liquid interface. Therefore, these cells can form a stratified squamous epithelium in vitro. The epidermis, which constitutes the upper part of the skin, is continuously renewed by proliferating keratinocytes, and can hence be divided into different layers: (1) the basal layer, or stratum basale, contains proliferating keratinocytes that differentiate as they move up in the epidermis; (2) in the spinous layer, or stratum spinosum, the keratinocytes reinforce their cytoskeleton and interact with one another via desmosomes; (3) the granular layer, or stratum granulosum, contains flat anuclear keratinocytes with numerous proteins, such as filaggrins, which aggregate in granules; (4) the stratum corneum is the outermost layer, and is formed by dead keratinocytes or corneocytes sealed together by secreted lipids and rich in keratin filaments [25,26]. Recently, a novel in vitro system using 3D organotypic raft cultures to study MCPyV-associated MCC was reported [27]. In this model, when the MCPyV^+^ MCC MKL1 cell line was embedded in a collagen layer placed between the dermal equivalent (containing human fibroblasts) and the epidermal equivalent (comprising human keratinocytes), MCC-like lesions arose within the dermal equivalent independently of the presence of keratinocytes. However, co-culture of MCPyV^+^ MCC and keratinocytes within the epidermal equivalent did not reproduce the morphology of human MCC, as MKL1 cells did not survive within such a raft [27].

In the present study, we established OERCs of MCC by using virus-positive and virus-negative MCC cell lines. OERCs of transformed cell lines have proven to be useful for investigating the growth and pathogenesis of DNA viruses, for testing antiviral compounds, and for studying tumor growth [28,29]. Our OERCs were characterized histologically and immunohistochemically, demonstrating that they are a suitable tool for studying MCC growth and evaluating the efficacy and selectivity of successful drug candidates previously assayed in monolayer cell cultures. Moreover, gene expression analysis of a panel of growth factors and cell adhesion molecules revealed differences in the expression profiles of tumor cells and normal human keratinocytes at the transcriptional level.

## 2. Materials and Methods

### 2.1. Cell Culture

The present study involving human cell lines was conducted according to the principles expressed in the Declaration of Helsinki. All cell lines were obtained and used as approved by the Belgian equivalent of the International Review Board (Departement Leefmilieu, Natuur en Energie, protocol SBB 219 2011/0011) and the Biosafety Committee at KU Leuven. The WAGA cell line was kindly provided by Roland Houben (University Hospital Würzburg, Germany). The other MCC cell lines were obtained from the European Collection of Authenticated Cell Cultures (ECACC): MCC13 (Cat#10092302), MCC14/2 (Cat#10092303), MCC26 (Cat#10092304), MS-1 (Cat#09111802), and MKL-1 (Cat#09111801). MCPyV^−^ MCC cell lines (MCC13, MCC14/2, and MCC26) were grown in RPMI 1640 medium supplemented with 10% fetal bovine serum (FBS). MCPyV^+^ MCC cell lines (MS-1, MKL-1, and WAGA) were maintained in RPMI 1640 + GlutaMAX^TM^-l medium supplemented with 20% FBS. Primary human keratinocytes (PHKs) derived from human foreskin biopsies were routinely obtained from Hôpitaux Iris Sud (Brussels, Belgium). Briefly, biopsies were washed with 70% ethanol and Dulbecco’s phosphate-buffered saline (DPBS) supplemented with 2.5 µg/mL amphotericin B and 50 µg/mL gentamicin. Fragmented biopsies were digested in trypsin–EDTA (0.25%) for 1.5 h at 37 °C. The obtained cell suspension was passed through a 70 µm cell strainer and plated in keratinocyte–SFM (serum-free medium) medium supplemented with human recombinant epidermal growth factor (EGF) and bovine pituitary extract (BPE). 3T3-J2 murine fibroblasts—a kind gift from Thomas Broker (University of Alabama, Tuscaloosa, AL, USA)—were grown in DMEM medium supplemented with 10% FBS. Media supplements (1× MEM NEAA, 1 mM sodium pyruvate, 1× penicillin/streptomycin/glutamine, and 10 mM HEPES) were added to RPMI, DMEM, and keratinocyte–SFM media. All media and supplements were from Thermo Fisher Scientific (Merelbeke, Belgium).

### 2.2. Generation of Organotypic Epithelial Raft Cultures

**Figure 1:**
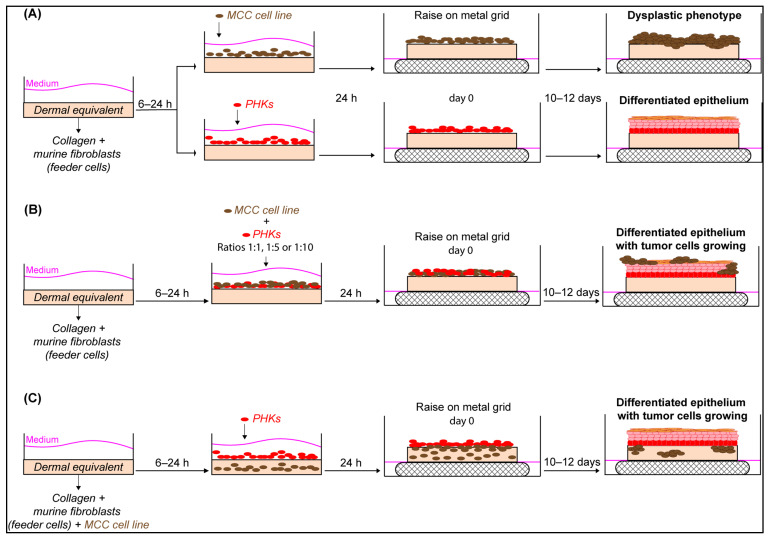
Development of organotypic epithelial raft cultures of Merkel cell carcinoma cell lines. Different strategies were assayed for the development of organotypic epithelial raft cultures (OERCs). (**A**) In the first option, a matrix or dermal equivalent was prepared by mixing type 1 rat-tail collagen with murine fibroblasts, which functioned as feeder cells, in a well of a 24-well plate. Dermal equivalents were covered with medium and left to equilibrate for 6–24 h in the incubator. Then, the cell lines of choice—a determined MCC cell line or primary human keratinocytes (PHKs)—were seeded on top of the dermal equivalent and incubated at 37 °C with 5% CO_2_. The next day, dermal equivalents were lifted on stainless metal grids so that cell cultures could grow at the air–liquid interface for 10–12 days. Eventually, tumor cells were expected to show a dysplastic phenotype, while PHKs should differentiate in a stratified epithelium resembling human skin. (**B**) Dermal equivalents were prepared as described above. Then, PHKs and MCC cells were mixed at different ratios and seeded on top of the dermal equivalents. In this case, tumor cells were expected to proliferate in the stratified epithelium formed by the differentiation of PHKs. (**C**) MCC cells were also included in the matrix mixture for the preparation of the dermal equivalents. Once equilibrated, PHKs were added, and cultures were allowed to differentiate. Tumor cells were expected to proliferate in the dermal equivalent, whereas PHKs were expected to differentiate.

**Figure 2:**
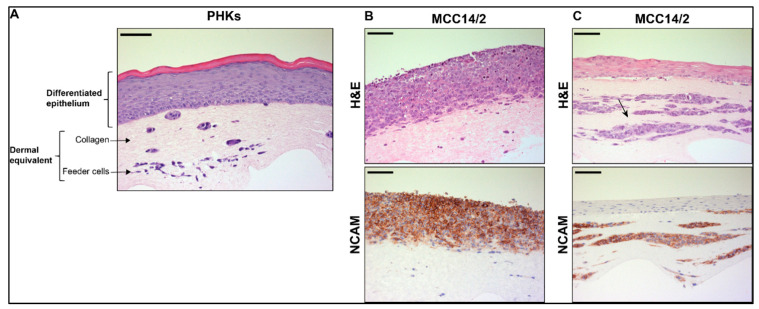
Organotypic epithelial raft cultures (OERCs) of primary human keratinocytes and tumor cells. (**A**) Hematoxylin and eosin (H&E)-stained section of an OERC of primary human keratinocytes (PHKs). The dermal equivalent is composed of collagen, and contains murine fibroblasts used as feeder cells. On top of the dermal equivalent, the PHKs form a differentiated epithelium. (**B**) The MCPyV^−^ tumor cell line MCC14/2 proliferates on top of the dermal equivalent, showing a dysplastic phenotype, without the presence of PHKs, as observed in the H&E-stained section. These cells express the MCC marker NCAM. (**C**) MCC14/2 cells also proliferate in OERCs when embedded into the dermal equivalents, as indicated by the arrow, while the PHKs form a differentiated epithelium on top. MCC14/2 cells growing into the dermal equivalent also express NCAM. All images were taken at an overall 200× magnification, and the bars equal 100 μm.

**Figure 3:**
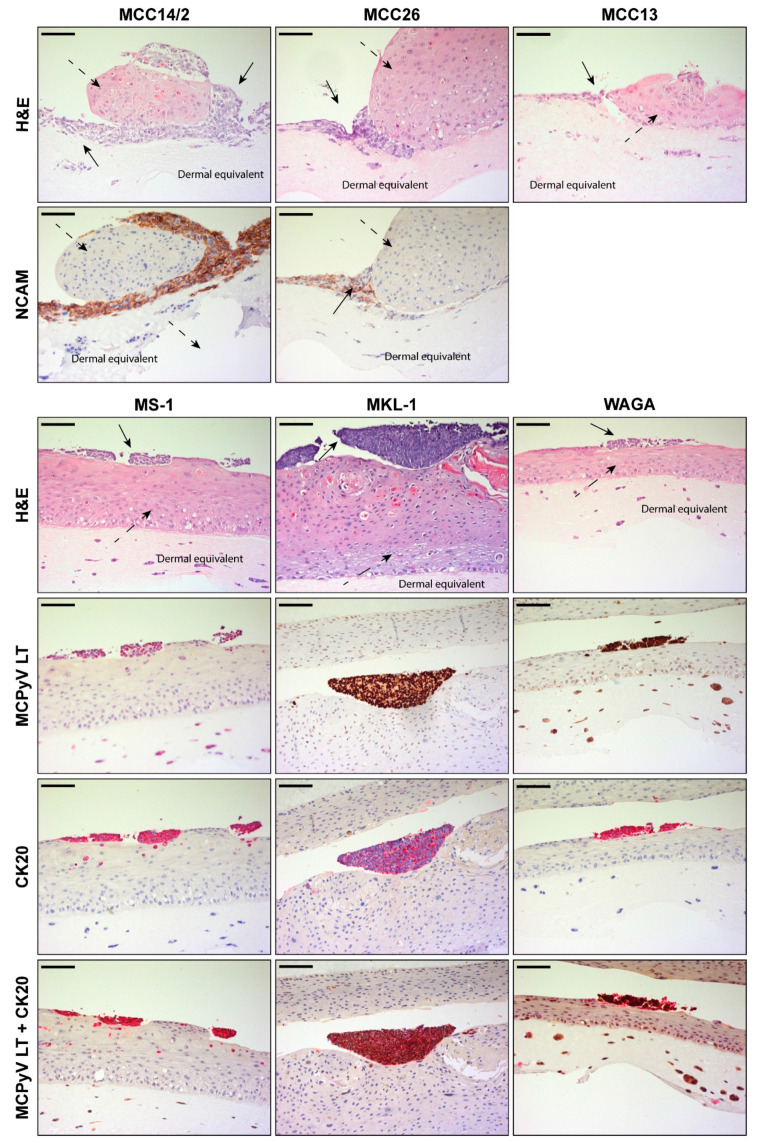
Histological analysis of organotypic epithelial raft cultures (OERCs) of MCC cell lines co-cultured with primary human keratinocytes (PHKs). MCPyV^−^ MCC cell lines (MCC14/2, MCC26 and MCC13) grow in OERCs (indicated by arrows in the H&E sections) when co-cultured with PHKs at a ratio of 1 to 5. Except MCC13, these cell lines express NCAM, a typical MCC marker. MCPyV^+^ MCC cell lines (MS-1, MKL-1 and WAGA) also proliferate in our 3-D culture model, as indicated by full arrows in the H&E sections, when co-cultured with PHKs. These cells express the LT of MCPyV (although the murine fibroblasts stain positive as well, likely due to cross-reactivity to the murine antibody) and the MCC marker CK20, as confirmed by IHC. Double IHC staining for MCPyV LT (brown) and CK20 (red) show co-localization of these signals. PHKs differentiate in a normal epithelium (pointed out by dashed arrows). All images were taken at 20× magnification and the bars equal 100 μm.

Three different approaches were investigated for the development of OERCs of MCC:

(1) Cells grown on a dermal equivalent: Firstly, a matrix mixture for the generation of dermal equivalents was prepared on ice by mixing type 1 rat-tail collagen (Thermo Fisher Scientific), 10× concentrated Ham’s F12 nutrient mix (Thermo Fisher Scientific), 10× reconstitution buffer (2.2% NaHCO_3_, 0.05 N NaOH and 200 mM HEPES buffer), and 1–2 × 10^5^ feeder cells (3T3-J2 fibroblasts). Subsequently, 900 µL of the matrix mixture were poured into each well of 24-well plates. After 1 h, 1 mL of Dulbecco–F12 medium (3:1 DMEM to Ham’s F12 nutrient mix with media supplements and 10% FBS) was added, and dermal equivalents were left to equilibrate for 6–24 h in a humidified incubator with 5% CO_2_ at 37 °C. Next, Dulbecco–F12 medium was removed, and 2 × 10^5^ cells from an MCC cell line (or PHKs) in 1:1 Dulbecco–F12 to 10%/20% RPMI (or keratinocyte–SFM) were seeded on top of the dermal equivalent and incubated for 24 h at 37 °C (Figure 1A);
(2) Co-cultures of MCC/PHKs grown on a dermal equivalent: Dermal equivalents were prepared as described above. PHKs and MCC cells were mixed at different ratios, added on top of the dermal equivalents, and incubated in 1:1:1 Dulbecco–F12 to 10%/20% RPMI and keratinocyte–SFM for 24 h at 37 °C (Figure 1B);
(3) MCC cells incorporated into the dermal equivalent: Fibroblasts were included in the matrix mixture to which MCC cells were added (2 × 10^5^ MCC cells were included in the matrix mixture for the preparation of the dermal equivalent). Following 8 h incubation with 1:1 Dulbecco–F12 medium to 10%/20% RPMI medium, 2 × 10^5^ PHKs were added, and cultures were further incubated in 1:1:1 Dulbecco–F12 to 10%/20% RPMI and keratinocyte–SFM for 24 h at 37 °C (Figure 1C).

Following 24 h of incubation submerged in culture medium, dermal equivalents were lifted on a stainless metal grid at the air–liquid interface. Cultures were incubated in raft culture medium (1:1 Dulbecco–F12 to keratinocyte–SFM, additionally supplemented with 5 µg/mL of insulin and 0.4 µg/mL of hydrocortisone), which was replaced every 3 days. Raft cultures were allowed to grow for the desired time (usually 10–12 days) at 37 °C. Then, raft cultures were harvested by fixing them in 10% buffered formalin, or were kept in the freezer, depending on the need for further analysis.

### 2.3. Histological and Immunohistochemical Analysis

Raft cultures were harvested, fixed in 10% buffered formalin, and embedded in paraffin. Deparaffinized sections were stained with hematoxylin and eosin (H&E) for histological examination at the Laboratory of Translational Cell and Tissue Research, Department of Imaging and Pathology, KU Leuven. H&E staining was performed on the Autostainer XL with Coverslipper (Leica Biosystems, Wetzlar, Germany). For immunohistochemistry (IHC), antigen epitope retrieval was performed with either Tris/EDTA (H2) or citrate (H1) buffer. Subsequently, IHC was executed in a Bond Max Autostainer (Leica Biosystems) using a BOND Polymer Refine Detection Kit (Cat#DS9800 from Leica), with the following primary antibodies: anti-MCPyV LT (Cat#sc-136172) and anti-matrix metalloproteinase 13 (MMP13) (Cat#sc-515284) from Santa Cruz Biotechnology (Dallas, TX, USA); anti-cytokeratin 20 (CK20) (Cat#IR777) from Agilent Technologies (Diegem, Belgium); anti-neural cell adhesion molecule (NCAM) (Cat#3576) and anti-cleaved caspase 3 (Cat#9661) from Cell Signaling Technology (Danvers, MA, USA); anti-Ki67 (Cat#ab833), anti-bone morphogenic protein 7 (BMP7) (Cat#ab56023), and anti-matrix metalloproteinase 9 (MMP9) (Cat#ab76003) from Abcam (Cambridge, UK); and anti-fibroblast growth factor 13 (FGF13) (Cat#PA5-27302) from Thermo Fisher Scientific. Pictures of the stained sections were taken with a Zeiss Axio Imager Z1 microscope and analyzed using Axio Vision v.4 (Carl Zeiss AG, Jena, Germany).

### 2.4. RNA Extraction and cDNA Synthesis

First, frozen rafts were allowed to thaw; then, they were disrupted and homogenized with QIAzol Lysis Reagent (Qiagen, Benelux BV, Antwerpen, Belgium). Briefly, 700 µL of QIAzol was added to the Eppendorf tube containing the whole raft, which was homogenized by vortexing for 1 min. Next, the tube was placed on the benchtop for 15 min. Upon addition of 140 µL of chloroform, RNA extraction was performed with the RNeasy Mini Kit (Qiagen), following the manufacturer’s instructions, and including a DNase digestion step with the RNase-Free DNase Set (Qiagen) to digest any contaminant DNA. The product was eluted in 30 µL of RNase-free water, and the RNA concentration was determined by A260 reading with a NanoDrop ND-1000 Spectrophotometer (Isogen Life Science, Sint-Pieters-Leeuw, Belgium). The extracted RNA was stored frozen at −80 °C until further analysis.

The High-Capacity cDNA Reverse Transcription Kit with RNase inhibitor (Thermo Fisher Scientific) was used for cDNA synthesis, following the manufacturer’s instructions. Reactions were run on an Eppendorf Mastercycler Pro S (Eppendorf, Hamburg, Germany) with the following program: 25 °C for 10 min, 37 °C for 120 min, and 85 °C for 5 min, with a final hold step at 4 °C. cDNA concentration was assessed using a NanoDrop ND-1000 Spectrophotometer and diluted to 20 ng/µL (Isogen Life Science).

### 2.5. TaqMan Arrays

TaqMan Array Plates (Thermo Fisher Scientific), in a fast 96-well plate format, were used to investigate the gene expression profiles of human growth factors (Cat#4418802) and human extracellular matrix and adhesion molecules (Cat#4418778) in our OERCs of MCC cell lines. Detailed information of the composition of each panel of molecules is provided in Tables S1 and S2. Briefly, the cDNA samples were combined with 2× TaqMan Fast Universal PCR Master Mix (Thermo Fisher Scientific), and 10 µL of this mix was placed in each well of the TaqMan Array Plate. In this array, target cDNA was amplified using sequence-specific primers and 5′-FAM dye-labelled TaqMan MGB probes. Reactions were run on an ABI 7500 Fast Real-Time PCR System (Applied Biosystems, Foster City, CA, USA). Cycling conditions were as follows: 50 °C for 2 min, 95 °C for 20 s, followed by 40 cycles of 95 °C for 3 s and 60 °C for 30 s. For each of the two arrays used, three plates were run for each raft type. Data were analyzed using 7500 Fast System SDS software v1.4 (Applied Biosystems) and normalized to the endogenous control GAPDH (glyceraldehyde 3-phosphate dehydrogenase). Then, mRNA expression was quantified relative to that of OERCs of PHKs grown on top of the dermal equivalents, using the 2^−ΔΔCt^ method [30].

### 2.6. Statistics

Each TaqMan array was repeated at least three times to calculate the mean ± SD values of each target gene. Multiple *t*-test analysis was performed with GraphPad Prism 8 (GraphPad Software Inc., La Jolla, CA, USA) to compare the expression levels of each target gene between the different 3D cultures. Significance was defined as *p* < 0.05.

## 3. Results

### 3.1. MCC Cells, except MCC14/2, Required Co-Culture with Keratinocytes to Proliferate in OERCs

OERCs were generated as described in the Materials and Methods section (Figure 1). At day 10 or 12 post-lifting, OERCs were fixed and subsequently subjected to histological analysis. Hematoxylin and eosin (H&E) staining of OERCs derived from PHKs seeded on top of dermal equivalents showed that these cells were able to proliferate and form a differentiated epithelium resembling the normal epithelium seen in vivo (Figure 2A), as previously reported [28]. However, MCC14/2 cells—the only MCC cell line that did not require the support of PHKs to proliferate in our 3D culture model—proliferated, forming a layer of highly atypical cells (Figure 2B). The results essentially point to one cell line (MCC14/2) growing in all three types of raft, and the WAGA, MS-1, and MKL-1 MCPyV^+^ MCC cells and the MCC13 and MCC26 MCPyV^−^ MCC cells only growing when mixed with the PHK cells in the epithelial layer (Table 1).

IHC staining of the rafts derived from MCC14/2 cells with an antibody against NCAM—a marker of MCC—confirmed the identity of these cells, and did not label surrounding keratinocytes or murine fibroblasts in the dermal equivalent (Figure 2B and Figure S1). However, staining of MCC14/2 was negative for other common markers of MCC, such as CK20, chromogranin A, and CK7. MCC14/2 was also the only MCC cell line able to grow when embedded into the dermal equivalent, as shown by NCAM expression, which allowed their distinction from murine feeder cells (Figure 2C and Figure S1).

As illustrated in Figure 3 and Figure S2, all MCC cell lines proliferated to different extents in OERCs when co-cultured with PHKs at different ratios (1:1, 1:5, and 1:10). Considering that at a 1:5 ratio of tumor cells to normal keratinocytes, all MCPyV^+^ and MCPyV^−^ MCC cell lines showed a reasonable growth without replacing the PHKs or being scarcely distributed in the rafts, further experiments were carried out using a 1:5 ratio. H&E and IHC analysis of MCC14/2 in co-culture with PHKs showed prominent growth of tumor cells, which hampered the formation of a differentiated epithelium by the PHKs, though rarely infiltrating into it (Figure 3). The same occurred with MCC26 and MCC13, although they only grew in close contact with PHKs. Therefore, the displacement of the keratinocytes was detrimental to these two tumor cell lines. Regarding MCC13 cells, IHC was negative for NCAM and other MCC markers, such as CK20, CK7, and chromogranin A.

MCPyV^+^ MCC cell lines co-cultured with PHKs in OERCs displayed a different growth pattern when compared with the negative counterparts. Generally, MS-1, MKL-1, and WAGA cells proliferated on top of the differentiated epithelium, with scarce infiltration in the underlying layers. The tumor cells stained positive upon IHC staining with an antibody against MCPyV LT (CM2B4), although the antibody showed cross-reactivity as the murine fibroblasts of the dermal equivalent were diffusively stained. However, CK20 exclusively stained the tumor cells. Furthermore, MCPyV LT and CK20 positivity co-localized upon double IHC staining. Specific detection of MCPyV LT showed intense nuclear localization, except for WAGA cells, while the cytoplasm stained positive for CK20.

### 3.2. OERCs of MCC Cells Exhibited Expression of Both Proliferative and Apoptotic Markers

Additional IHC staining with proliferation (Ki67) and apoptosis (cleaved caspase 3) markers was performed at different time points to investigate the formation of the differentiated epithelium, the kinetics of growth of tumor cells, and the interaction of both tumor cells and PHKs when co-cultured in OERCs. Therefore, OERCs of PHKs alone, MCC14/2 alone, co-cultures of MCC14/2 with PHKs, and co-cultures of MKL-1 with PHKs were fixed at different days after lifting on the metal grid.

**Figure 4:**
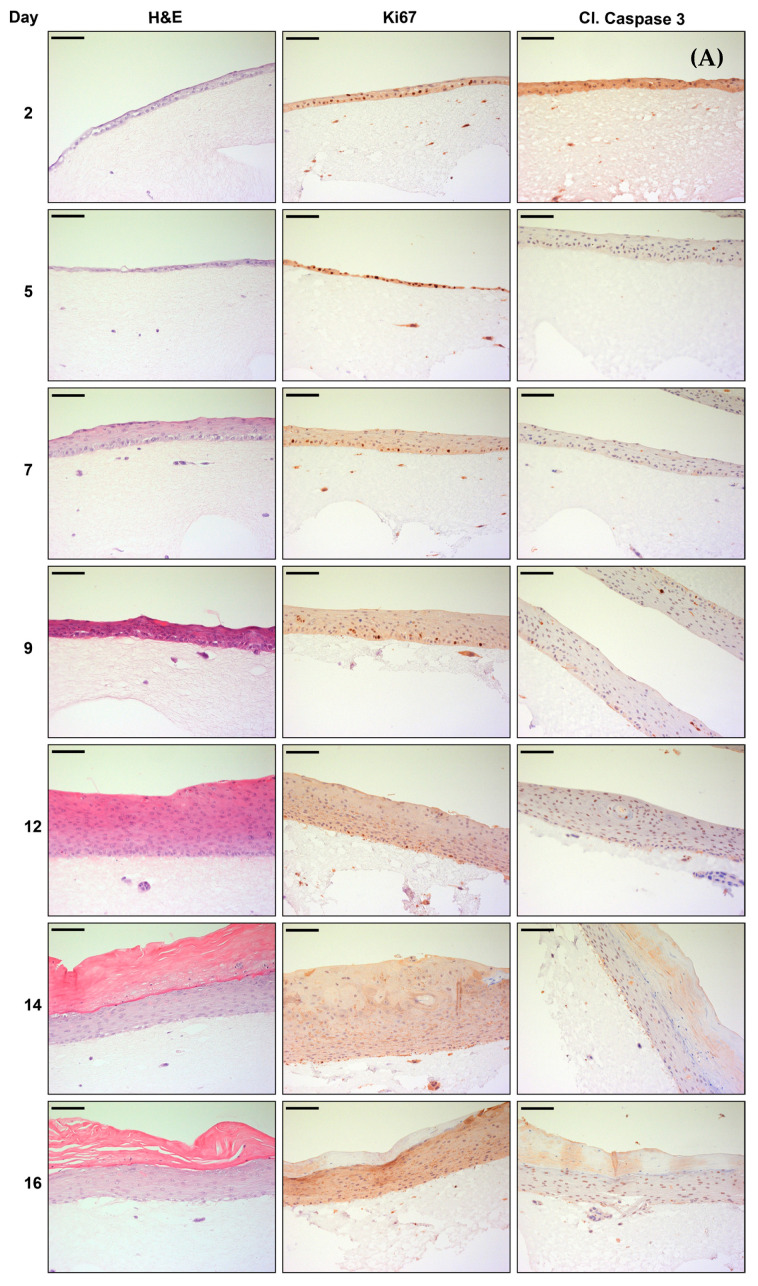

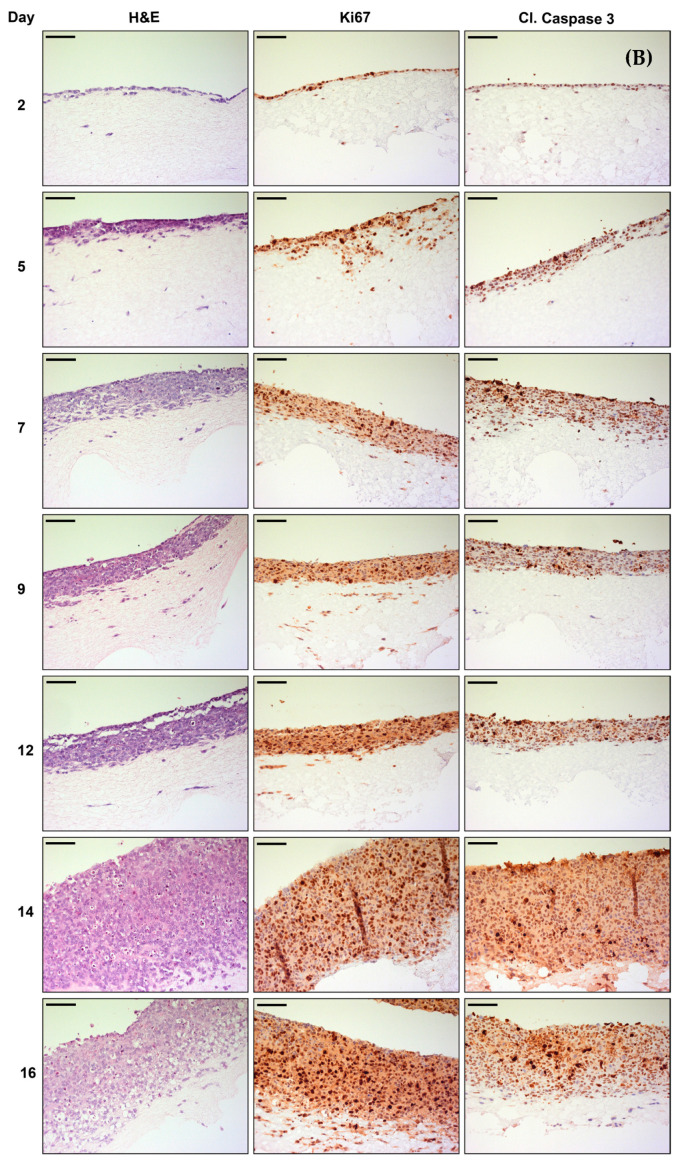
Morphological analysis of organotypic epithelial raft cultures (OERCs) of (**A**) PHKs and (**B**) MCC14/2 over time. Hematoxylin and eosin (H&E) staining and immunohistochemical (Ki67 and cleaved caspase 3 staining) analysis were performed at different time points after lifting the rafts. All images were taken at an overall 200× magnification, and the bars equal 100 μm.

H&E staining in Figure 4A shows the growth and differentiation of PHKs, which formed a stratified epithelium with abundant keratin at days 14 and 16 after lifting of the OERCs. As expected, PHKs stained with the proliferation marker Ki67 were normally restricted to the basal layers of the epithelium, and were more abundant at the early stages of differentiation. Apoptosis appeared to be rare, according to the scarce positivity for the apoptosis marker cleaved caspase 3. Histological analysis of OERCs of the MCPyV^−^ cell line MCC14/2 revealed sustained proliferation of the tumor cells over time, with abundant infiltration in the dermal equivalent (Figure 4B). Indeed, the tumor cells were strongly positive for Ki67. Contrary to the OERCs of PHKs, IHC analysis of cleaved caspase 3 in rafts of MCC14/2 resulted in marked staining of the tumor cells.

**Figure 5:**
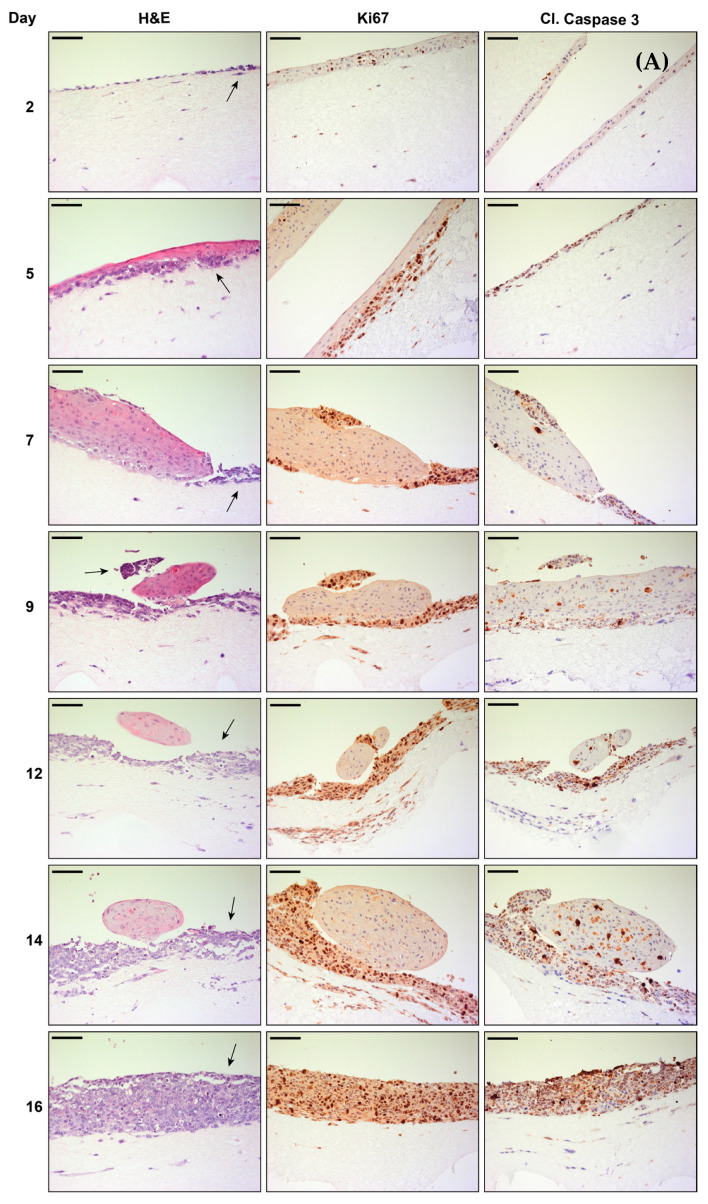

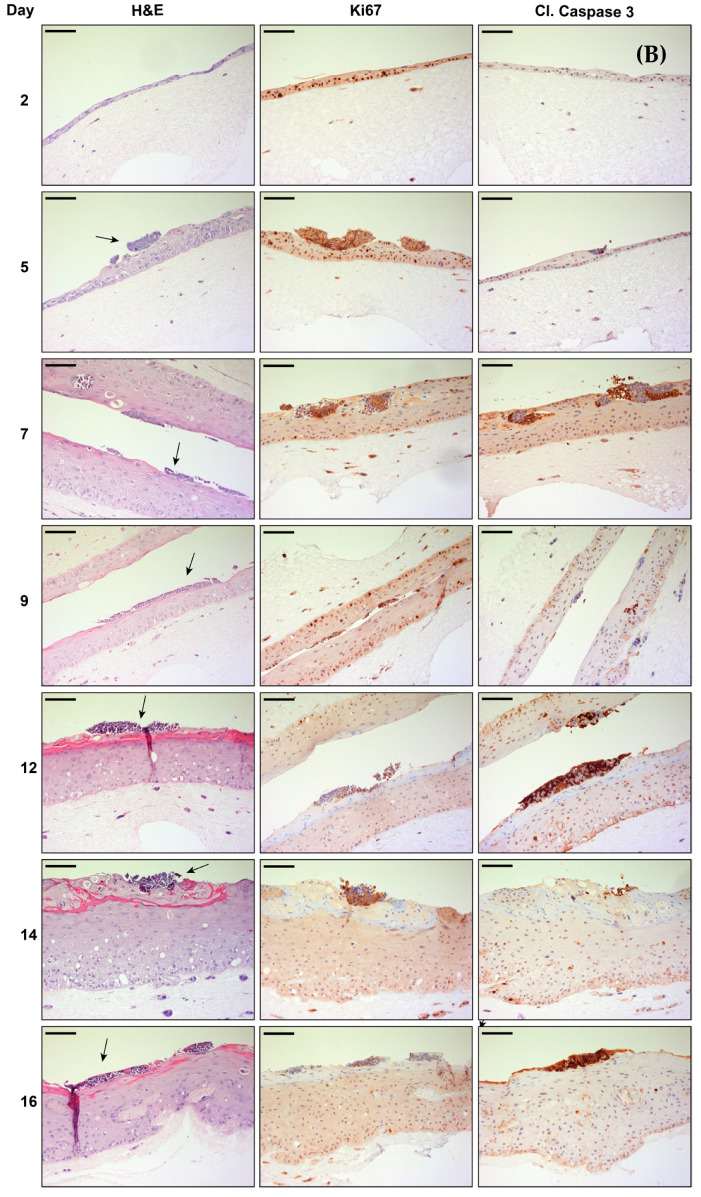
Morphological analysis of organotypic epithelial raft cultures (OERCs) of tumor cells co-cultured with primary human keratinocytes (PHKs) over time. (**A**) Hematoxylin and eosin (H&E) staining and immunohistochemical (Ki67 and cleaved caspase 3 staining) analysis of MCC14/2 cells (indicated by arrows in the H&E sections) co-cultured with PHKs at different time points after lifting the rafts. (**B**) H&E staining and immunohistochemical (Ki67 and cleaved caspase 3 staining) analysis of MKL-1 cells (indicated by arrows in the H&E sections) co-cultured with PHKs at different time points after lifting the rafts. All images were taken at an overall 200× magnification, and the bars equal 100 μm.

Co-cultures of MCC14/2 with PHKs showed how the tumor cells displaced the PHKs as they kept proliferating until day 16—the last time point investigated (Figure 5A). This was confirmed by the intense staining for Ki67 in the tumor cells. Furthermore, cleaved caspase 3 staining was more abundant in PHKs co-cultured with MCC14/2 than when grown alone, because of less contact with feeder cells. Nevertheless, as illustrated in Figure 5B, the MCPyV^+^ cell line MKL-1 behaved differently when co-cultured with PHKs in the OERC model. Generally, as with other MCPyV^+^ cell lines, the tumor cells only slightly proliferated on top of the stratified epithelium. Ki67 positivity revealed prominent proliferation of tumor cells until day 9 post-lifting. However, staining for Ki67 was lost in tumor cells as the stratum corneum of the epithelium formed. Cleaved caspase 3 staining was markedly intense in the MKL-1 tumor cells—especially from day 12 post-lifting onwards. 

### 3.3. Gene Expression Analysis of OERCs

As described above, several MCC cell lines behaved differently when grown in the OERC model. To gain insight into the molecular mechanisms involved in these distinct growth patterns, gene expression analysis of a panel of genes coding for extracellular matrix and cell adhesion molecules (panel 1, Table S1) and another panel of genes coding for growth factors (panel 2, Table S2) was assessed by qPCR using predesigned TaqMan array plates. This analysis was performed on frozen whole rafts, from which RNA was isolated for gene expression analysis, meaning that the extracted RNA was derived from both PHKs and MCC cells, as well as from the murine fibroblasts in the dermal equivalent.

**Figure 6:**
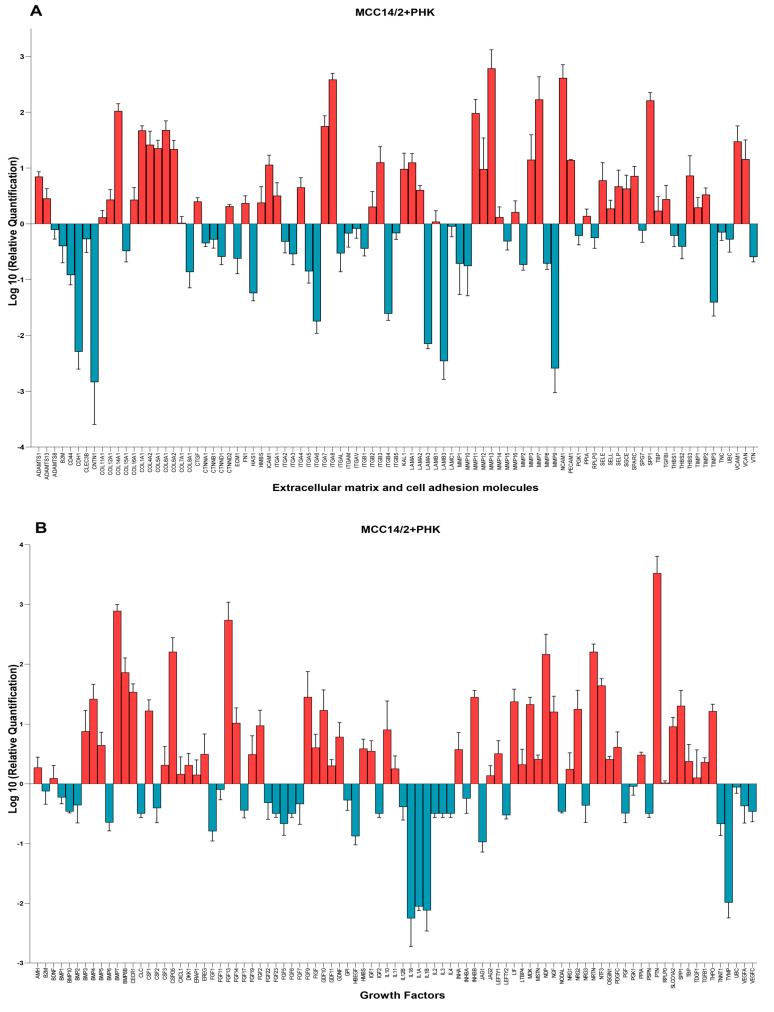
Gene expression profiles of co-cultures of MCC14/2 cells with primary human keratinocytes (PHKs). The plots depict the fold-change expression of (**A**) extracellular matrix and cell adhesion molecules and (**B**) growth factors relative to their expression in OERCs of PHKs. Data represent mean values ± SD of three independent experiments.

Since MCC14/2 was the only cell line capable of growing in the absence of PHKs, we first analyzed the gene expression of a panel of genes in OERCs of MCC14/2 cells grown alone or in the presence of PHKs. The expression, at the transcriptional level, of genes from panels 1 and 2 in OERCs of MCC14/2 cells co-cultured with PHKs (Figure 6A,B, respectively) and in OERCs of MCC14/2 (Figure S3), was calculated as the fold-change increase relative to the expression of the same genes in OERCs of PHKs.

Comparison between the expression profiles of OERCs of MCC14/2 cells when grown in co-culture and those of MCC14/2 grown alone did not reveal significant differences, indicating that there were no significant differences in the expression of the selected genes (coding for extracellular matrix and cell adhesion molecules and for growth factors) when PHKs were present. The expression profiles of the selected genes for OERCs of MCPyV^+^ cells (MS-1, MKL-1, and WAGA) co-cultured with PHKs, calculated as the fold-change increase relative to the expression of the same genes in OERCs of PHKs, are depicted in Figures S4–S6, respectively. The 10 most highly expressed genes from panel 1 in OERCs of MCC14/2 (grown alone or in co-culture with PHKs) and of MCPyV^+^ cell lines co-cultured with PHKs relative to PHK rafts are represented in Table 2.

Significant differences (*p* < 0.05) were observed when comparing the expression levels of genes from panel 1 in co-cultures of PHKs with MCC14/2 versus co-cultures of PHKs with MS-1 (CD44, CNTN1, COL14A1, COL15A1, COL1A1, CTNND2, ICAM1, ITGA6, ITGA8, ITGB4, LAMA3, LAMB3, MMP13, MMP9, PECAM1, SPP1, VCAM1), MKL-1 (COL1A1, COL6A1, HAS1, LAMA2), and WAGA (CD44, CDH1, CNTN1, COL15A1, COL1A1, COL5A1, ICAM1, ITGA6, ITGA8, ITGB2, LAMA3, LAMB3, MMP13, MMP3, MMP9, PECAM1, SPARC, THBS3).

Table 3 shows the 10 most highly expressed genes from panel 2 (coding for growth factors) in the same types of OERCs. In these cases, significant differences (*p* < 0.05) were also observed when comparing the expression levels in co-cultures of PHKs with MCC14/2 versus co-cultures of PHKs with MS-1 (NDP, NRTN, TYMP), MKL-1 (BMP7, PTN), and WAGA (BMP4, IL1A, NDP, PPIA, PTN, THPO).

**Figure 7:**
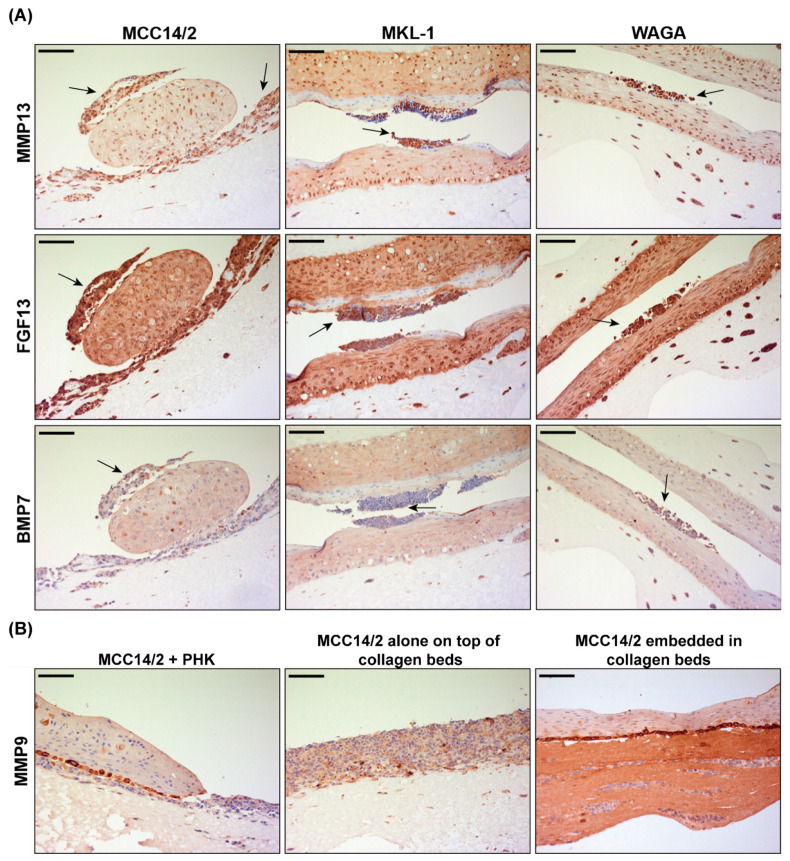
Immunohistochemical analysis of organotypic epithelial raft cultures (OERCs). (**A**) MCC14/2, MS-1, MKL-1, or WAGA cells co-cultured with primary human keratinocytes (PHKs) expressing matrix metalloproteinase 13 (MMP13), fibroblast growth factor 13 (FGF13), and bone morphogenic protein 7 (BMP7), as indicated by arrows. (**B**) Matrix metalloproteinase 9 (MMP9) is highly expressed by proliferating basal keratinocytes, and occasionally by MCC14/2 cells. All images were taken at an overall 200× magnification, and the bars equal 100 μm.

To validate a selected panel of genes identified as being differentially expressed in the MCC OERCs, IHC was performed on frozen whole rafts. Since RNA was extracted from all cell types present in the rafts—including PHKs, MCC cells, and fibroblasts—IHC analysis was meant to serve as verification of differentially expressed genes specifically in MCC cells, and to help in identifying which of the three cell types contribute to the gene signature identified in their gene expression analysis. The presence of some of the most highly expressed genes included in panels 1 and 2 according to the microarray experiments—such as matrix metalloproteinase 13 (MMP13), fibroblast growth factor 13 (FGF13), and bone morphogenic protein 7 (BMP7)—in the rafts of tumor cells (MCC14/2, MKL-1, and WAGA) co-cultured with PHKs, was investigated by IHC (Figure 7A). The IHC data indicate that the MCC cells in the raft co-cultures expressed these three genes and contributed to their differential expression in the co-cultures.

Conversely, PHKs exhibited a marked expression of MMP9 (Figure 7B)—especially in the basal layer of proliferating keratinocytes. Nevertheless, MCC14/2 cells also expressed MMP9, although the expression was focal and at a lower level than in the PHKs. For instance, MCC14/2 cells embedded into the dermal equivalent exhibited numerous granules containing MMP9 (Figure 7B).

## 4. Discussion

MCC is an aggressive type of skin cancer for which the current treatment approaches are limited. Therefore, there is an urgent need to explore new therapeutic options [1]. In this regard, an in vitro model that closely mimics MCC’s biology is strongly desirable. Thus far, most preclinical assays have been limited to monolayer cell cultures of MCC cell lines and xenografts in immunodeficient mice [11,12]. Here, we present a 3D cell culture model of MCC where the cells are interconnected between them and the extracellular matrix (ECM), resembling the physiological conditions more closely than monolayer cell cultures [31].

As previously shown, PHKs differentiated into a stratified epithelium when seeded on top of an artificial dermis, and with the support of murine fibroblasts [32]. Cells present in OERCs received nutrients and growth factors from the culture medium via diffusion through the dermal equivalent. Among all of the MCC cell lines tested, only MCC14/2 cells proliferated without the support of PHKs in OERCs when seeded on and when embedded into dermal equivalents. All other cell lines required co-culture with PHKs. However, MCC26 and MCC13 proliferation overtook the growth of PHKs and, consequently, their own growth was limited. Interestingly, MCPyV^+^ cells were almost exclusively present on top of the stratified epithelium. The differences in the behavior of virus-positive and virus-negative cells could be explained by their characteristic growth patterns: MCPyV^+^ cells grow as aggregates or single-cell suspensions, while the virus-negative cells are adherent [15]. Histological analysis showed that the OERCs contained small, round cells with marginal cytoplasm and round nuclei—general features of MCC tumors [1]. Except for MCC13, tumor cell lines grown in OERCs expressed markers of MCC, such as NCAM, CK20 or, in the case of the MCPyV^+^ cells, the viral LT. Furthermore, Ki67 staining confirmed tumor growth (MCC14/2 and MKL-1 cells) in OERCs. Nevertheless, while MCC14/2 cells were highly proliferative and exhibited a balance between cell growth and apoptosis, MKL1 cells reduced their growth and markedly stained for cleaved caspase 3 after 12 days of culture. Given their location on top of the keratinized epithelium, limited access to nutrients could explain the cell death. Our findings are consistent with a previous report concluding that the MKL1 cell line does not survive within the epithelial equivalent of the raft [27].

Gene expression analysis of OERCs with two different panels revealed increased expression of several genes with important functions in tumor growth, invasion, and metastasis. IHC confirmed the expression of MMP13 or collagenase 3, which is involved in degradation of the ECM and has been related to tumor angiogenesis [33]. Another highly expressed MMP, MMP7, has been associated with poor prognosis in MCC [34]. OERCs were also evaluated for expression of FGF13, which has been related to cancer cell survival and tumor metastasis [35,36]. BMP7, which has been shown to influence proliferation and cancer cell invasion [37], exhibited focal expression in our OERCs. MMP9, highly expressed by basal PHKs in our OERCs, has been shown to promote terminal differentiation of keratinocytes [38].

When comparing the expression profiles of OERCs of MCC14/2 alone with those of MCC14/2 in co-culture with PHKs, no significant differences were detected. Therefore, we hypothesized that PHKs did not significantly influence the gene expression profile of MCC14/2—at least not for the analyzed genes. However, several genes were differently expressed when co-cultures of MCC14/2 cells were compared with co-cultures of MCPyV^+^ cells. Co-cultures of MCC14/2 cells showed increased expression of various collagen genes (*COL*), which may explain the prominent proliferation of this cell line in our 3D culture model. Indeed, overexpression of some of these genes has been related to tumor progression and metastasis [39]. Other upregulated genes (e.g., *THBS3*, *SPARC*, and *SPP1*) have also been implicated in cancer cell invasiveness [40]. Several genes coding for cell adhesion molecules (CAMs), which are involved in tumor angiogenesis (i.e., *ICAM1*, *PECAM1*, and *VCAM1*), were highly expressed by OERCs of MCC cells—especially MCC14/2 [41]. Furthermore, the most highly transcribed growth factor in OERCs of MCC14/2—pleiotrophin (*PTN*)—was reported to be involved in angiogenesis and tumor progression [42]. Finally, a growth factor expressed by all of the OERCs of tumor cells—i.e., neurotrophin-3 (*NTF3*)—was related to brain metastasis in breast cancer [43]. Importantly, these results should be taken with caution. The transcriptional expression levels corresponded to the pool of all cells present in the raft, which in some cases included PHKs, tumor cells, and murine fibroblasts. Although the presence of RNA from feeder cells may have been marginal in OERCs of PHKs and MCC14/2 cells grown alone, it would be desirable to analyze the expression profile of each cell type individually—especially in the OERCs of co-cultures. Here, we used IHC to validate the expression of three genes—*FGF3*, *MMP13*, and *BMP7*—in MCC cells in the MCC + PHK co-cultures. This means that tumor cells contributed to the higher expression of these genes in the co-cultures, which is consistent with the function of the proteins encoded by these genes. Fibroblast growth factor 13 (FGF13) is a member of the fibroblast growth factor family, and FGF family members have broad mitogenic and cell survival activities. Upregulated expression of FGF13 contributes to tumor growth, tumor invasion, and resistance to platinum drugs in some cancers [35,36,44,45]. Upregulated expression of matrix metalloproteinase 13 (MMP13) is known to enhance metastasis and invasion [46,47,48]. Bone morphogenetic protein 7 (BMP7)—one of the members of the BMP family of signaling molecules—is implicated in various types of cancer, influencing the proliferation, migration, and invasion of cancer cells [37,49,50]. Our data suggest a potential role of FGF13, MMP13, and BMP7 in the biology of MCC—both MCPyV^+^ and MCPyV^−^—which should be further explored. Future experiments should also compare PHK–MCC cell monolayer co-cultures to rafted/co-cultured PHK–MCC cells to unravel how gene expression patterns change when cultured in a three-dimensional setting.

Lambert et al. recently reported on the development of a novel in vitro 3D culture system for the co-culture of the MKL-1 (MCPyV^+^) cell line [27]. MCPyV^+^ MCC cells embedded in a collagen layer between the epidermal equivalent (comprising human keratinocytes) and a dermal equivalent (containing human fibroblasts) were identified as the optimal culture conditions, leading to MCC-like lesions arising within the dermal equivalent. When MCPyV^+^ MCC cells were co-cultured together with keratinocytes within the epidermal equivalent of the raft, human MCC morphology could not be reproduced, as MKL1 cells did not survive within such a raft. Furthermore, Loke et al. [27] found that keratinocytes were not necessary for the formation of MCC-like lesions in the dermal equivalent. Although we and Loke et al. both used a 3D culture system, their findings and ours differ, which could be explained by differences in the methodology and in the setup of the rafts. Unlike Lambert’s study, we used primary human keratinocytes (PHKs) rather than a spontaneously immortalized human keratinocyte cell line (NIKS), and their dermal equivalent contained early-passage human foreskin fibroblasts (EF-1F), while we used the J2 immortalized murine fibroblasts. Future studies should be directed towards evaluating the optimal setup reported by Loke et al., comparing the three MCPyV^+^ (MS-1, MKL-1, and WAGA) and the three MCPyV^−^ (MCC13, MCC14/, and MCC26) cell lines used in the present study. In contrast to Loke et al., we found that PHKs were necessary for the growth of MCCs (except for MCC14/2), which can be ascribed to differences in the cells used in the dermal equivalent (murine fibroblasts versus human fibroblasts), as well as to the duration of the experiments before harvesting the rafts (10–12 days for our experiments versus ~29 days in Loke’s work). Moreover, important setup differences should be highlighted, as we did not use an intermediate layer with MCC cells placed between the dermal equivalent and the epidermal equivalent, which was Loke’s optimal culture condition. In our experiments, it is intriguing that none of the MCC cell lines except one (MCC14/2) grew when embedded in the dermis, despite MCC being considered a cancer that arises most frequently in the dermis. However, it has been recently proposed that MCC may arise from two different cells of origin: MCPyV^−^ MCC from epidermal keratinocytes, and MCPyV^+^ MCC from dermal fibroblasts [51]. Future epigenetic experiments are required in order to confirm the distinct lineages of the MCPyV^+^ and MCPyV^−^ MCC^−^ subtypes. It should be mentioned that under our experimental conditions, differences between MCC cell lines could be observed, pointing to the importance of employing various MCC cell lines that may have acquired some adaptation after passage in cell culture.

Compared to animal models, in vitro 3D cell culture models are less costly, easy to manipulate, require shorter timeframes, and are adaptable for higher throughput studies—though this latter aspect remains challenging [52]. Furthermore, results obtained in experimental animals are not always translatable to the clinic, due to physiological divergences, such as different gene expression in murine and human skin [53]. Although most MCC cell lines grew in OERCs under our conditions, the current model presents certain limitations. First, tumor cell lines do not always recapitulate the molecular heterogeneity of MCC. Moreover, only two of the used cell lines (MKL-1 and WAGA) have been argued to be representative of MCCs, and the origin of certain MCPyV^−^ cell lines is still questionable [15]. Nevertheless, OERCs are suitable to investigate tumor growth and response to drugs, but standardization across experiments and adaptation to high-throughput analysis are still needed. Second, MCCs are frequently found in the dermis, but only MCC14/2 cells grew when embedded into the dermal equivalents [1]. The use of collagen produced by human fibroblasts—which conserves the particular structure and post-translational modifications—and replacement of the murine fibroblasts with human neonatal fibroblasts to mimic the native human stroma may facilitate the growth of MCC cell lines in OERCs [54]. Finally, our model lacks minority cell types of the epidermis, such as Langerhans cells, melanocytes, and Merkel cells. Furthermore, the vascular and immune systems are absent in the dermal equivalents.

Other models, such as CDXs, have their own strengths and limitations for predicting drug efficacy [55]. Although PDXs closely mimic the tumor of origin, and are useful to predict clinical responses, the tumor microenvironment is replaced by murine components [56]. Furthermore, they require immunodeficient mice, although efforts are being made to generate humanized animals with competent immune cells [57]. Larger timeframes for animal breeding also hamper the process of preclinical testing [58]. Recent advances, such as microfluidic devices, have allowed the development of more physiologically relevant models with the introduction of new factors—such as oxygen and nutrient gradients as well as perfusable vasculature with endothelial cells—to increase their clinical predictive value [52]. Moreover, modern 3D printing technology enables the creation of tissues with varying degree of complexity. Different cell types—such as pericytes, fibroblasts, and endothelial cells—can be printed with a defined spatial pattern to mimic the tumor stroma [59]. Although the inclusion of vasculature is crucial for the integration of other cell types of the skin—such as hair, sweat glands, nerves, and immune cells—this remains challenging [60].

More than half of drugs under investigation fail in clinical trials due to lack of efficacy or safety issues. Hence, the importance of introducing preclinical models that better recapitulate in vivo tumor biology [52]. OERCs of MCC cell lines present a great opportunity for preclinical therapeutic testing; they provide a unique means of investigating the efficacy and specificity of new drug candidates and drug combinations against MCC, with the potential to rapidly identify drug resistance. Furthermore, they can be adapted to the culture of patient-derived organoids, which preserve the tumor microenvironment—including functional immune cells—and genetic heterogeneity [61]. OERCs may also be used to study biological aspects of MCC, such as the cell of origin and tumorigenesis, whether virus-related or UV-light-induced. In fact, the study of human papillomavirus (HPV) in OERCs has been instrumental in identifying cellular factors involved in viral pathogenesis [62].

Further investigation of some of the most highly expressed genes in our OERCs may provide a new repertoire of biomarkers for MCC. Similarly, deregulated genes involved in metastatic tumor behavior or drug resistance might be identified and used as future therapeutic targets.

## 5. Conclusions

OERCs of MCC cell lines have the potential to be used as a powerful tool for evaluating the efficacy and selectivity of new drug candidates against MCC, facilitating the translation of basic research findings into clinical practice. This is of vital importance for this malignancy, considering the limited availability of patient samples owing to the rarity and aggressiveness of the disease. Moreover, gene expression analysis of rafts generated with distinct MCC cell lines revealed differences in the transcriptional profiles of the rafts containing tumor cells when compared with those generated exclusively with primary human keratinocytes.

## Figures and Tables

**Table 1:** Growth of MCPyV^+^ and MCPyV^−^ MCC cell lines under different experimental conditions in OERCs.

Culturing Conditions in the 3D Cultures	Results
(a) MCC cells added on top of the collagen beds (dermal equivalent) before lifting the rafts to the air–liquid interface	Only the MCPyV^−^ cell line MCC14/2 was able to grow
(b) MCC cells + PHKs mixed at different ratios added to the collagen beds (dermal equivalent) before lifting to the air–liquid interface	The three MCPyV^+^ (WAGA, MS-1, and MLK-1) and the three MCPyV^−^ (MCC13, MCC26, MCC14/2) cell lines were able to grow
(c) MCC cells embedded in the collagen beds	Only the MCPyV^−^ cell line MCC14/2 was able to grow

**Table 2:** Extracellular matrix and cell adhesion molecules highly expressed in OERCs of MCC cell lines. mRNA expression in each type of MCC OERC was quantified relative to the gene expression in OERCs of PHKs grown on top of the dermal equivalents, using the 2^−ΔΔCt^ method. Gene symbols for the 10 most expressed molecules in each MCC OERC relative to PHK rafts are indicated. Genes highly expressed in the five MCC OERC types are indicated in bold and in different colors.

Culture Condition	1	2	3	4	5	6	7	8	9	10
*MCC14/2 alone*	*MMP13*	***NCAM1***	*ITGA8*	*SPP1*	*MMP7*	*COL14A1*	***MMP11***	***ITGA7***	*COL1A1*	*COL6A1*
*Co-culture MCC14/2 + PHK*	*MMP13*	***NCAM1***	*ITGA8*	*MMP7*	*SPP1*	*COL14A1*	***MMP11***	***ITGA7***	*COL6A1*	*COL1A1*
*Co-culture MS-1 + PHK*	***NCAM1***	*CTNND2*	***MMP11***	***ITGA7***	*ITGB3*	*MMP16*	*MMP7*	*MMP3*	*HAS1*	*TGFBI*
*Co-culture MKL-1 + PHK*	*CTNND2*	*ADAMTS8*	***MMP11***	***ITGA7***	***NCAM1***	*PECAM1*	*VCAN*	*MMP8*	*MMP3*	*MMP13*
*Co-culture WAGA + PHK*	***NCAM1***	*SPP1*	*CTNND2*	*MMP16*	***ITGA7***	*MMP2*	***MMP11***	*MMP7*	*ITGB3*	*HAS1*

**Table 3:** Growth factors highly expressed in OERCs of MCC cell lines. mRNA expression in each type of MCC OERC was quantified relative to the gene expression in OERCs of PHKs grown on top of the dermal equivalents, using the 2^−ΔΔCt^ method. Gene symbols for the 10 most expressed molecules in each MCC OERC relative to PHK rafts are indicated. Genes highly expressed in the five MCC OERC types are indicated in bold and in different colors.

Culture Condition	1	2	3	4	5	6	7	8	9	10
*MCC14/2 alone*	*PTN*	***FGF13***	***BMP7***	*CSPG5*	*NDP*	*NRTN*	*BMP8B*	***FGF9***	***NTF3***	*INHBB*
*Co-culture MCC14/2 + PHK*	*PTN*	***BMP7***	***FGF13***	*NRTN*	*CSPG5*	*NDP*	*BMP8B*	***NTF3***	*CECR1*	***FGF9***
*Co-culture MS-1 + PHK*	***NTF3***	***FGF9***	***BMP7***	*SLCO1A2*	***FGF13***	*BMP8B*	*CSPG5*	*FIGF*	*FGF14*	*MDK*
*Co-culture MKL-1 + PHK*	*NRTN*	*FGF14*	***NTF3***	***FGF9***	*NODAL*	*FGF17*	***FGF13***	*PTN*	*FIGF*	***BMP7***
*Co-culture WAGA + PHK*	***BMP7***	***FGF13***	***FGF9***	***NTF3***	*BMP5*	*BMP8B*	*CSPG5*	*SPP1*	*NRTN*	*GPI*

## Data Availability

The data presented in this study are available in the article and Supplementary Materials.

## Supplementary Materials

The following are available online at https://www.mdpi.com/article/10.3390/cancers14041091/s1: Figure S1: Specificity of NCAM staining for MCC cells; Figure S2: Organotypic epithelial raft cultures (OERCs) of Merkel cell carcinoma (MCC) cell lines co-cultured with primary human keratinocytes (PHKs) at ratios of 1:1, 1:5, and 1:10; Figure S3: Gene expression profiles of organotypic epithelial raft cultures (OERCs) of MCC14/2 cells grown alone on top of the dermal equivalents; Figure S4: Gene expression profiles of co-cultures of MS-1 cells with primary human keratinocytes (PHKs); Figure S5: Gene expression profiles of co-cultures of MKL-1 cells with primary human keratinocytes (PHKs); Figure S6: Gene expression profiles of co-cultures of WAGA cells with primary human keratinocytes (PHKs); Table S1: Panel of genes present in the human extracellular matrix and adhesion molecules array plate; Table S2: Panel of genes present in the human growth factors array plate.
