# Supplementary material for: Organotypic Epithelial Raft Cultures as a Three-Dimensional In Vitro Model of Merkel Cell Carcinoma
Source: Cancers (Basel). 2022 Feb 21;14(4):1091. doi: 10.3390/cancers14041091 (PMC8870341; doi:10.3390/cancers14041091)
Supplement: Supplementary file 1 [file cancers-14-01091-s001.zip › cancers-1543133-supplementary.pdf]

Supplementary Materials: Organotypic Epithelial Raft Cultures as a New Three-Dimensional In Vitro Model of Merkel Cell Carcinoma

Arturo Temblador, Dimitrios Topalis, Joost van den Oord, Graciela Andrei and Robert Snoeck

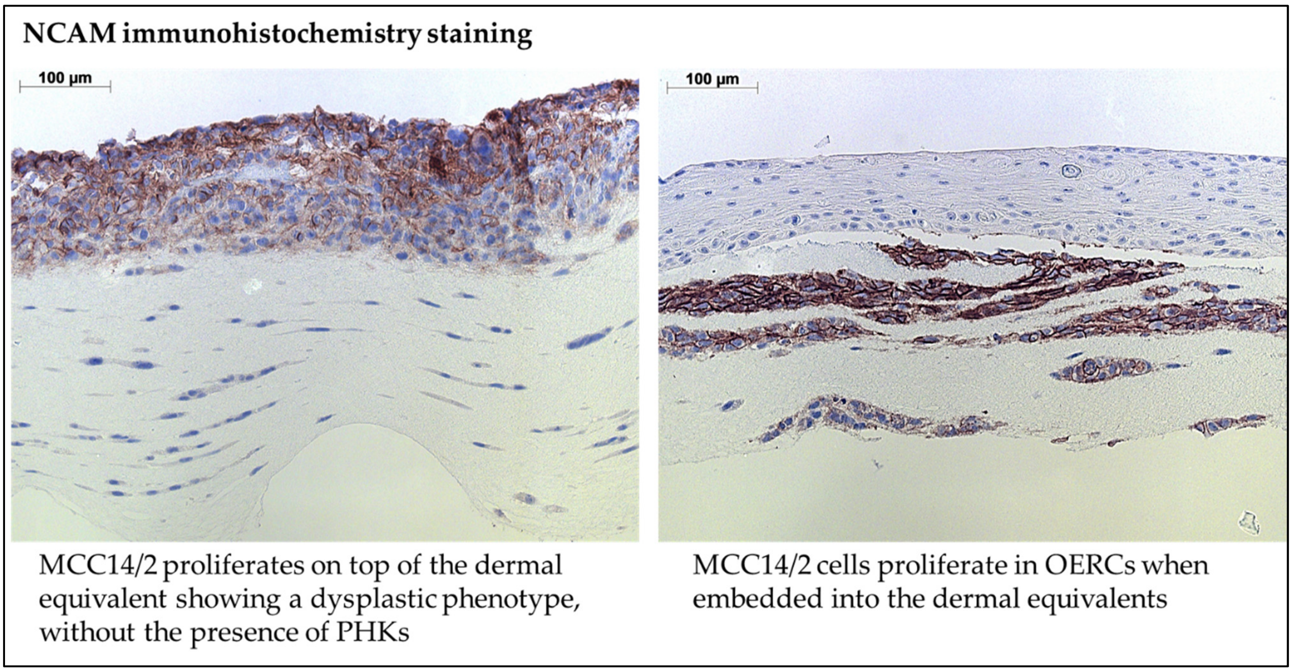

Figure S1. Specificity of NCAM staining for MCC cells. The MCPyV- MCC cell line MCC14/2 was grown either on top of the dermal equivalent (left image) or embedded into the dermal equivalent (right image). Murine fibroblasts in the dermal equivalent are clearly negative for NCAM. Images of the H&E staining were taken at an overal 200× magnification.

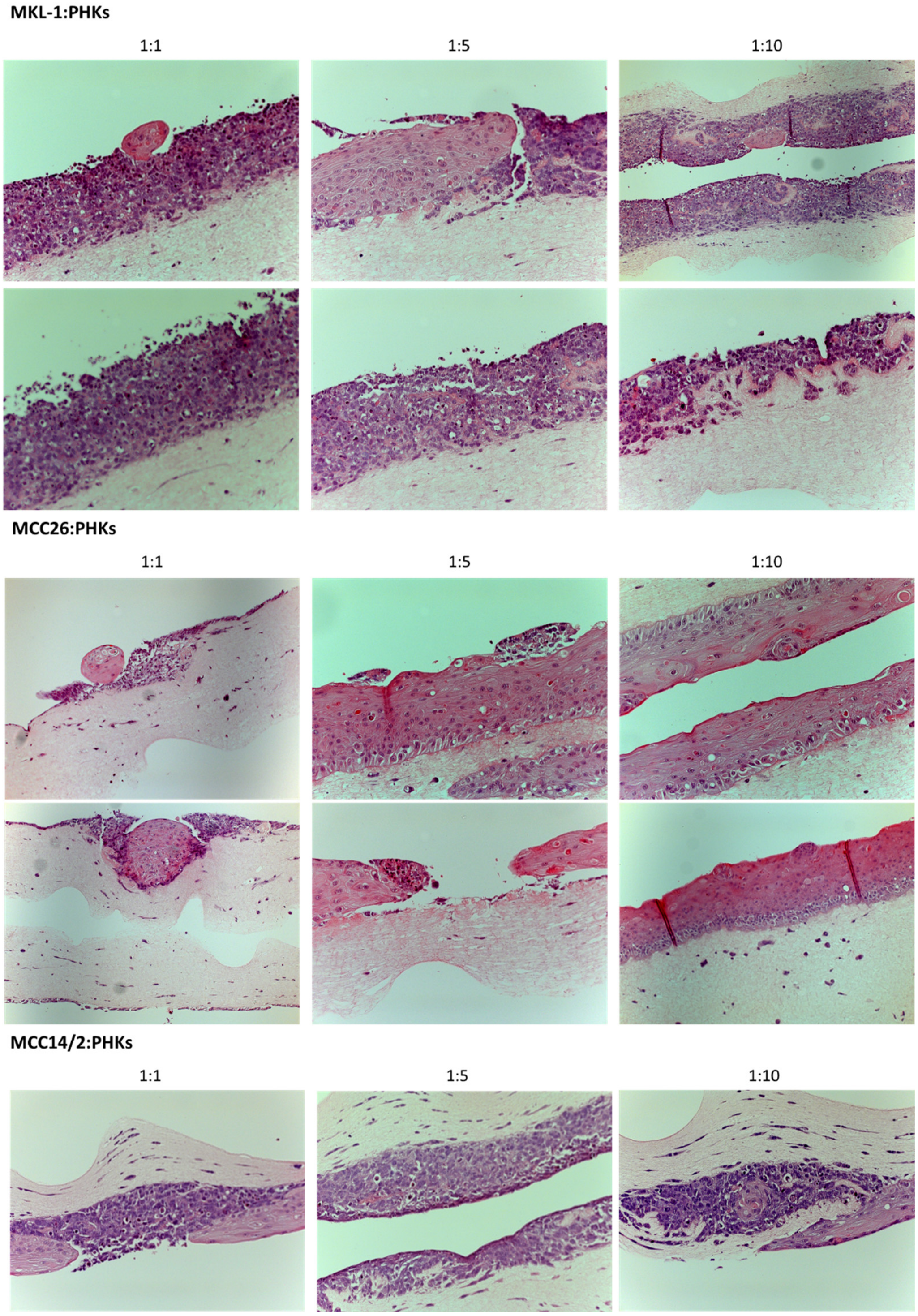
MKL-1:PHKs
1:1
1:5
1:10
MCC26:PHKs
1:1
1:5
1:10
MCC14/2:PHKs
1:1
1:5
1:10

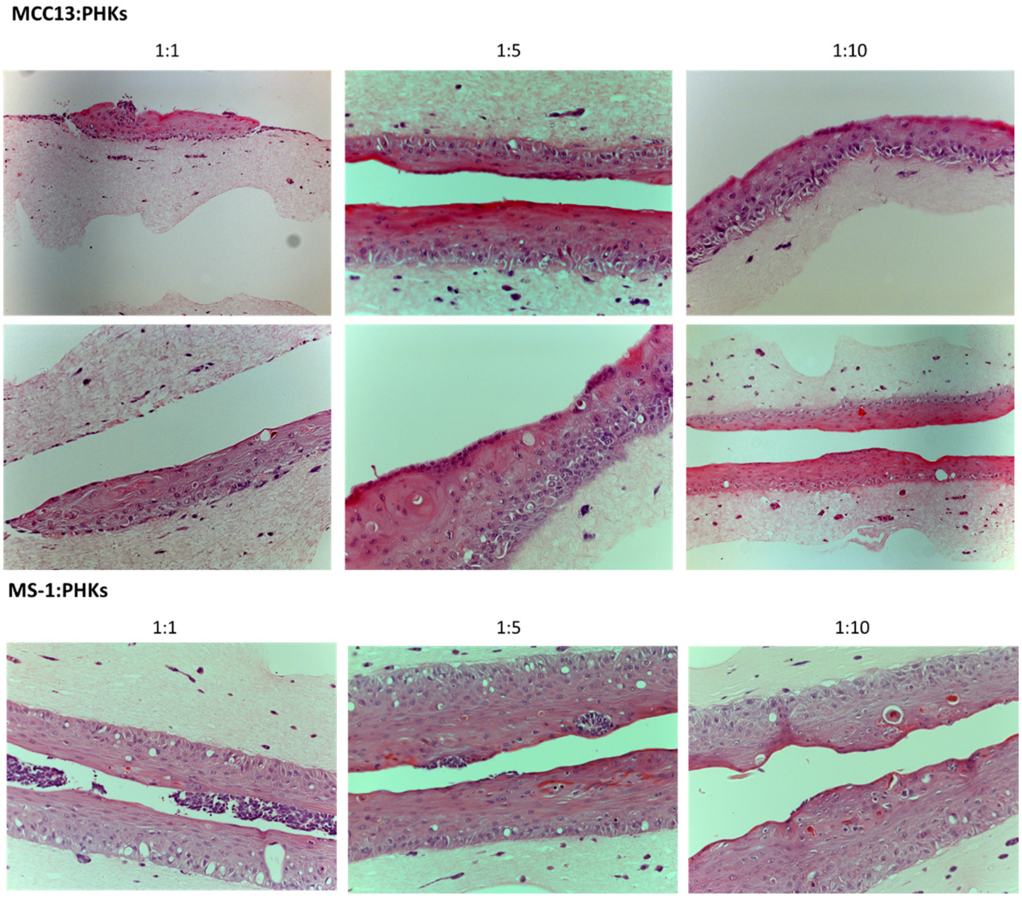

Figure S2. Organotypic epithelial raft cultures (OERCs) of Merkel cell carcinoma (MCC) cell lines co-cultures with primary human keratinocytes (PHKs) at a ratio of 1:1, 1:5 and 1:10. The MCPyV- MCC cell lines (MCC14/2, MCC26 and MCC13) and the MCPyV$^+$ MCC cell lines (MS-1, MKL-1 and WAGA) were used. Different strategies can be assayed for the development of organotypic epithelial raft cultures (OERCs). Images of the H&E staining were taken an overal 200× magnification.

Table S1. Panel of genes present in the Human Extracellular Matrix & Adhesion Molecules array plate. The description of each gene symbol is provided under the table.

	1	2	3	4	5	6	7	8	9	10	11	12
A	*18S*	*GAPDH*	*HPRT1*	*GUSB*	*ACTB*	*B2M*	*RPLP0*	*HMBS*	*TBP*	*PGK1*	*UBC*	*PPIA*
B	*ADAMTS1*	*ADAMTS13*	*ADAMTS8*	*CD44*	*CDH1*	*CNTN1*	*COL11A1*	*COL12A1*	*COL14A1*	*COL15A1*	*COL16A1*	*COL1A1*
C	*COL4A2*	*COL5A1*	*COL6A1*	*COL6A2*	*COL7A1*	*COL8A1*	*VCAN*	*CTGF*	*CTNNA1*	*CTNNB1*	*CTNND1*	*CTNND2*
D	*ECM1*	*FN1*	*HAS1*	*ICAM1*	*ITGA1*	*ITGA2*	*ITGA3*	*ITGA4*	*ITGA5*	*ITGA6*	*ITGA7*	*ITGA8*
E	*ITGAL*	*ITGAM*	*ITGAV*	*ITGB1*	*ITGB2*	*ITGB3*	*ITGB4*	*ITGB5*	*KAL1*	*LAMA1*	*LAMA2*	*LAMA3*
F	*LAMB1*	*LAMB3*	*LAMC1*	*MMP1*	*MMP10*	*MMP11*	*MMP12*	*MMP13*	*MMP14*	*MMP15*	*MMP16*	*MMP2*
G	*MMP3*	*MMP7*	*MMP8*	*MMP9*	*NCAM1*	*PECAM1*	*SELE*	*SELL*	*SELP*	*SGCE*	*SPARC*	*SPG7*
H	*SPP1*	*TGFBI*	*THBS1*	*THBS2*	*THBS3*	*TIMP1*	*TIMP2*	*TIMP3*	*CLEC3B*	*TNC*	*VCAM1*	*VTN*

**Gene Symbol**	**Description**
18S	Eukaryotic 18S rRNA
GAPDH	glyceraldehyde-3-phosphate dehydrogenase
HPRT1	hypoxanthine phosphoribosyltransferase 1
GUSB	glucuronidase, beta
ACTB	actin, beta
B2M	beta-2-microglobulin
RPLP0	ribosomal protein, large, P0
HMBS	hydroxymethylbilane synthase
TBP	TATA box binding protein

PGK1	phosphoglycerate kinase 1
UBC	ubiquitin C
PPIA	peptidylprolyl isomerase A (cyclophilin A)
ADAMTS1	ADAM metallopeptidase with thrombospondin type 1 motif, 1
ADAMTS13	ADAM metallopeptidase with thrombospondin type 1 motif, 13
ADAMTS8	ADAM metallopeptidase with thrombospondin type 1 motif, 8
CD44	CD44 molecule (Indian blood group)
CDH1	cadherin 1, type 1, E-cadherin (epithelial)
CNTN1	contactin 1
COL11A1	collagen, type XI, alpha 1
COL12A1	collagen, type XII, alpha 1
COL14A1	collagen, type XIV, alpha 1
COL15A1	collagen, type XV, alpha 1
COL16A1	collagen, type XVI, alpha 1
COL1A1	collagen, type I, alpha 1
COL4A2	collagen, type IV, alpha 2
COL5A1	collagen, type V, alpha 1
COL6A1	collagen, type VI, alpha 1
COL6A2	collagen, type VI, alpha 2
COL7A1	collagen, type VII, alpha 1
COL8A1	collagen, type VIII, alpha 1
VCAN	versican
CTGF	connective tissue growth factor
CTNNA1	catenin (cadherin-associated protein), alpha 1, 102kDa
CTNNB1	catenin (cadherin-associated protein), beta 1, 88kDa
CTNND1	catenin (cadherin-associated protein), delta 1
CTNND2	catenin (cadherin-associated protein), delta 2 (neural plakophilin-related arm-repeat protein)
ECM1	extracellular matrix protein 1
FN1	fibronectin 1
HAS1	hyaluronan synthase 1
ICAM1	intercellular adhesion molecule 1
ITGA1	integrin, alpha 1
ITGA2	integrin, alpha 2 (CD49B, alpha 2 subunit of VLA-2 receptor)
ITGA3	integrin, alpha 3 (antigen CD49C, alpha 3 subunit of VLA-3 receptor)
ITGA4	integrin, alpha 4 (antigen CD49D, alpha 4 subunit of VLA-4 receptor)
ITGA5	integrin, alpha 5 (fibronectin receptor, alpha polypeptide)
ITGA6	integrin, alpha 6
ITGA7	integrin, alpha 7
ITGA8	integrin, alpha 8
ITGAL	integrin, alpha L (antigen CD11A (p180), lymphocyte function-associated antigen 1; alpha polypeptide)
ITGAM	integrin, alpha M (complement component 3 receptor 3 subunit)
ITGAV	integrin, alpha V (vitronectin receptor, alpha polypeptide, antigen CD51)
ITGB1	integrin, beta 1 (fibronectin receptor, beta polypeptide, antigen CD29 includes MDF2, MSK12)
ITGB2	integrin, beta 2 (complement component 3 receptor 3 and 4 subunit)
ITGB3	integrin, beta 3 (platelet glycoprotein IIIa, antigen CD61)
ITGB4	integrin, beta 4
ITGB5	integrin, beta 5
KAL1	Kallmann syndrome 1 sequence
LAMA1	laminin, alpha 1

LAMA2	laminin, alpha 2
LAMA3	laminin, alpha 3
LAMB1	laminin, beta 1
LAMB3	laminin, beta 3
LAMC1	laminin, gamma 1 (formerly LAMB2)
MMP1	matrix metallopeptidase 1 (interstitial collagenase)
MMP10	matrix metallopeptidase 10 (stromelysin 2)
MMP11	matrix metallopeptidase 11 (stromelysin 3)
MMP12	matrix metallopeptidase 12 (macrophage elastase)
MMP13	matrix metallopeptidase 13 (collagenase 3)
MMP14	matrix metallopeptidase 14 (membrane-inserted)
MMP15	matrix metallopeptidase 15 (membrane-inserted)
MMP16	matrix metallopeptidase 16 (membrane-inserted)
MMP2	matrix metallopeptidase 2 (gelatinase A, 72kDa gelatinase, 72kDa type IV collagenase)
MMP3	matrix metallopeptidase 3 (stromelysin 1, progelatinase)
MMP7	matrix metallopeptidase 7 (matrilysin, uterine)
MMP8	matrix metallopeptidase 8 (neutrophil collagenase)
MMP9	matrix metallopeptidase 9 (gelatinase B, 92kDa gelatinase, 92kDa type IV collagenase)
NCAM1	neural cell adhesion molecule 1
PECAM1	platelet/endothelial cell adhesion molecule
SELE	selectin E
SELL	selectin L
SELP	selectin P (granule membrane protein 140kDa, antigen CD62)
SGCE	sarcoglycan, epsilon
SPARC	secreted protein, acidic, cysteine-rich (osteonectin)
SPG7	spastic paraplegia 7 (pure and complicated autosomal recessive)
SPP1	secreted phosphoprotein 1
TGFBI	transforming growth factor, beta-induced, 68kDa
THBS1	thrombospondin 1
THBS2	thrombospondin 2
THBS3	thrombospondin 3
TIMP1	TIMP metallopeptidase inhibitor 1
TIMP2	TIMP metallopeptidase inhibitor 2
TIMP3	TIMP metallopeptidase inhibitor 3
CLEC3B	C-type lectin domain family 3, member B
TNC	tenascin C
VCAM1	vascular cell adhesion molecule 1
VTN	vitronectin

Table S2. Panel of genes present in the Human Growth Factors array plate. The description of each gene symbol is provided under the table.

	1	2	3	4	5	6	7	8	9	10	11	12
**A**	*18S*	*GAPDH*	*HPRT1*	*GUSB*	*ACTB*	*B2M*	*RPLP0*	*HMBS*	*TBP*	*PGK1*	*UBC*	*PPIA*
**B**	*AMH*	*BDNF*	*BMP1*	*BMP10*	*BMP2*	*BMP3*	*BMP4*	*BMP5*	*BMP6*	*BMP7*	*BMP8B*	*CECR1*
**C**	*CLC*	*CSF1*	*CSF2*	*CSF3*	*CSPG5*	*CXCL1*	*DKK1*	*ERAP1*	*TYMP*	*EREG*	*FGF1*	*FGF11*
**D**	*FGF13*	*FGF14*	*FGF17*	*FGF19*	*FGF2*	*FGF22*	*FGF23*	*FGF5*	*FGF6*	*FGF7*	*FGF9*	*FIGF*
**E**	*GDF10*	*GDF11*	*MSTN*	*GDNF*	*GPI*	*HBEGF*	*IGF1*	*IGF2*	*IL10*	*IL11*	*IL12B*	*IL18*
**F**	*IL1A*	*IL1B*	*IL2*	*IL3*	*IL4*	*INHA*	*INHBA*	*INHBB*	*JAG1*	*JAG2*	*LEFTY1*	*LEFTY2*
**G**	*LIF*	*LTBP4*	*MDK*	*NDP*	*NGF*	*NODAL*	*NRG1*	*NRG2*	*NRG3*	*NRTN*	*NTF3*	*OSGIN1*

H	*PDGFC*	*PGF*	*PSPN*	*PTN*	*SLCO1A2*	*SPP1*	*TDGF1*	*TGFB1*	*THPO*	*TNNT1*	*VEGFA*	*VEGFC*

**Gene Symbol**	**Description**
18S	Eukaryotic 18S rRNA
GAPDH	glyceraldehyde-3-phosphate dehydrogenase
HPRT1	hypoxanthine phosphoribosyltransferase 1
GUSB	glucuronidase, beta
ACTB	actin, beta
B2M	beta-2-microglobulin
RPLP0	ribosomal protein, large, P0
HMBS	hydroxymethylbilane synthase
TBP	TATA box binding protein
PGK1	phosphoglycerate kinase 1
UBC	ubiquitin C
PPIA	peptidylprolyl isomerase A (cyclophilin A)
AMH	anti-Mullerian hormone
BDNF	brain-derived neurotrophic factor
BMP1	bone morphogenetic protein 1
BMP10	bone morphogenetic protein 10
BMP2	bone morphogenetic protein 2
BMP3	bone morphogenetic protein 3
BMP4	bone morphogenetic protein 4
BMP5	bone morphogenetic protein 5
BMP6	bone morphogenetic protein 6
BMP7	bone morphogenetic protein 7
BMP8B	bone morphogenetic protein 8b
CECR1	cat eye syndrome chromosome region, candidate 1
CLC	Charcot-Leyden crystal protein
CSF1	colony stimulating factor 1 (macrophage)
CSF2	colony stimulating factor 2 (granulocyte-macrophage)
CSF3	colony stimulating factor 3 (granulocyte)
CSPG5	chondroitin sulfate proteoglycan 5 (neuroglycan C)
CXCL1	chemokine (C-X-C motif) ligand 1 (melanoma growth stimulating activity, alpha)
DKK1	dickkopf homolog 1 (Xenopus laevis)
ERAP1	endoplasmic reticulum aminopeptidase 1
TYMP	thymidine phosphorylase
EREG	epiregulin
FGF1	fibroblast growth factor 1 (acidic)
FGF11	fibroblast growth factor 11
FGF13	fibroblast growth factor 13
FGF14	fibroblast growth factor 14
FGF17	fibroblast growth factor 17
FGF19	fibroblast growth factor 19
FGF2	fibroblast growth factor 2 (basic)
FGF22	fibroblast growth factor 22
FGF23	fibroblast growth factor 23
FGF5	fibroblast growth factor 5
FGF6	fibroblast growth factor 6
FGF7	fibroblast growth factor 7 (keratinocyte growth factor)
FGF9	fibroblast growth factor 9 (glia-activating factor)

FIGF	c-fos induced growth factor (vascular endothelial growth factor D)
GDF10	growth differentiation factor 10
GDF11	growth differentiation factor 11
MSTN	myostatin
GDNF	glial cell derived neurotrophic factor
GPI	glucose phosphate isomerase
HBEGF	heparin-binding EGF-like growth factor
IGF1	insulin-like growth factor 1 (somatomedin C)
IGF2	insulin-like growth factor 2 (somatomedin A)
IL10	interleukin 10
IL11	interleukin 11
IL12B	interleukin 12B (natural killer cell stimulatory factor 2, cytotoxic lymphocyte maturation factor 2, p40)
IL18	interleukin 18 (interferon-gamma-inducing factor)
IL1A	interleukin 1, alpha
IL1B	interleukin 1, beta
IL2	interleukin 2
IL3	interleukin 3 (colony-stimulating factor, multiple)
IL4	interleukin 4
INHA	inhibin, alpha
INHBA	inhibin, beta A
INHBB	inhibin, beta B
JAG1	jagged 1 (Alagille syndrome)
JAG2	jagged 2
LEFTY1	left-right determination factor 1
LEFTY2	left-right determination factor 2
LIF	leukemia inhibitory factor (cholinergic differentiation factor)
LTBP4	latent transforming growth factor beta binding protein 4
MDK	midkine (neurite growth-promoting factor 2)
NDP	Norrie disease (pseudoglioma)
NGF	nerve growth factor (beta polypeptide)
NODAL	nodal homolog (mouse)
NRG1	neuregulin 1
NRG2	neuregulin 2
NRG3	neuregulin 3
NRTN	neurturin
NTF3	neurotrophin 3
OSGIN1	oxidative stress induced growth inhibitor 1
PDGFC	platelet derived growth factor C
PGF	placental growth factor
PSPN	persephin
PTN	pleiotrophin
SLCO1A2	solute carrier organic anion transporter family, member 1A2
SPP1	secreted phosphoprotein 1
TDGF1	teratocarcinoma-derived growth factor 1
TGFB1	transforming growth factor, beta 1
THPO	thrombopoietin
TNNT1	troponin T type 1 (skeletal, slow)
VEGFA	vascular endothelial growth factor A
VEGFC	vascular endothelial growth factor C

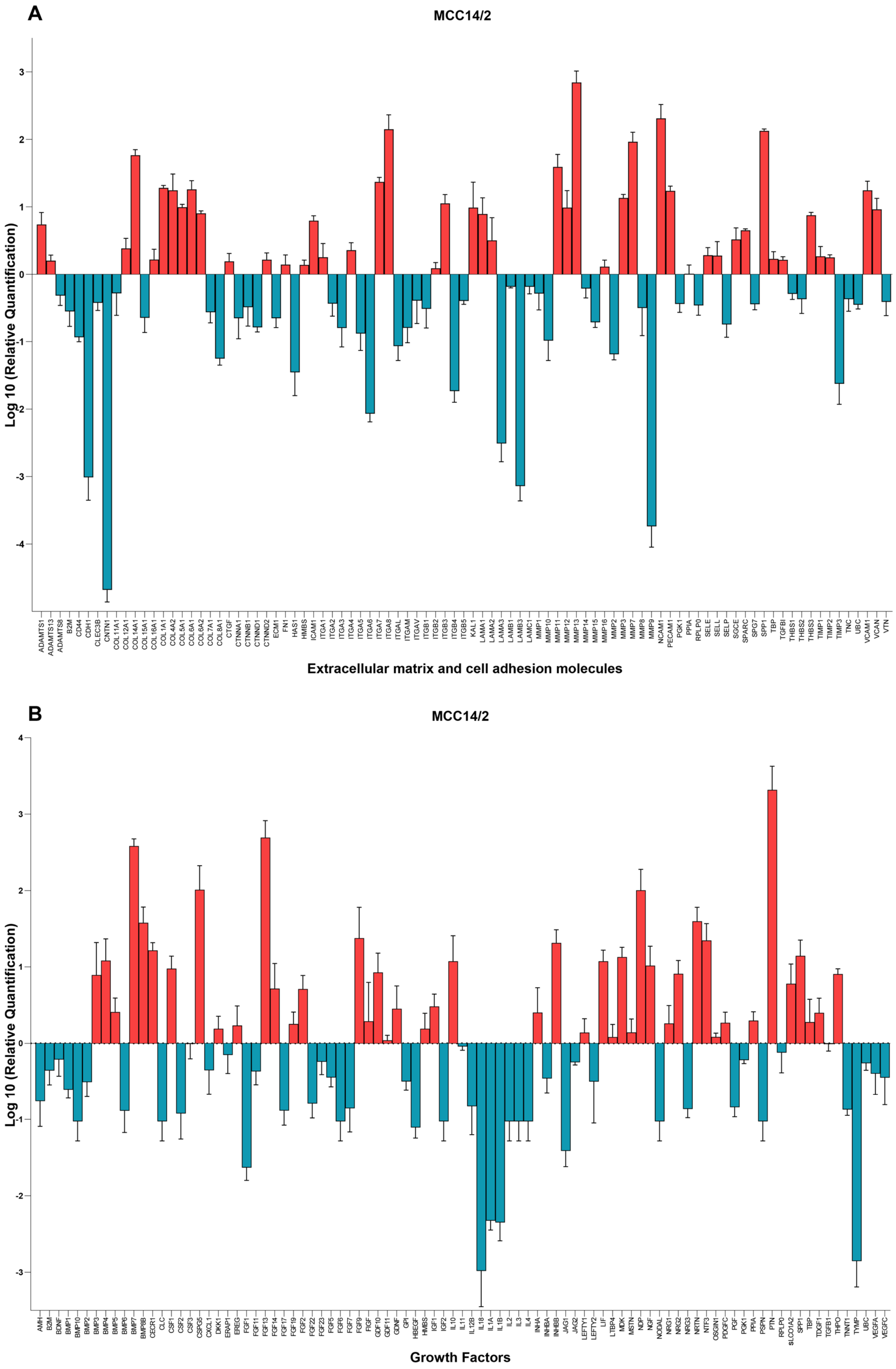

Figure S3. Gene expression profiles of organotypic epithelial raft cultures (OERCs) of MCC14/2 cells grown alone on top of the dermal equivalents. The plots depict the fold-change expression of (**A**) extracellular matrix and cell adhesion molecules and (**B**) growth factors relative to their expression in OERCs of PHKs. Data represent mean values ± SD of three independent experiments.

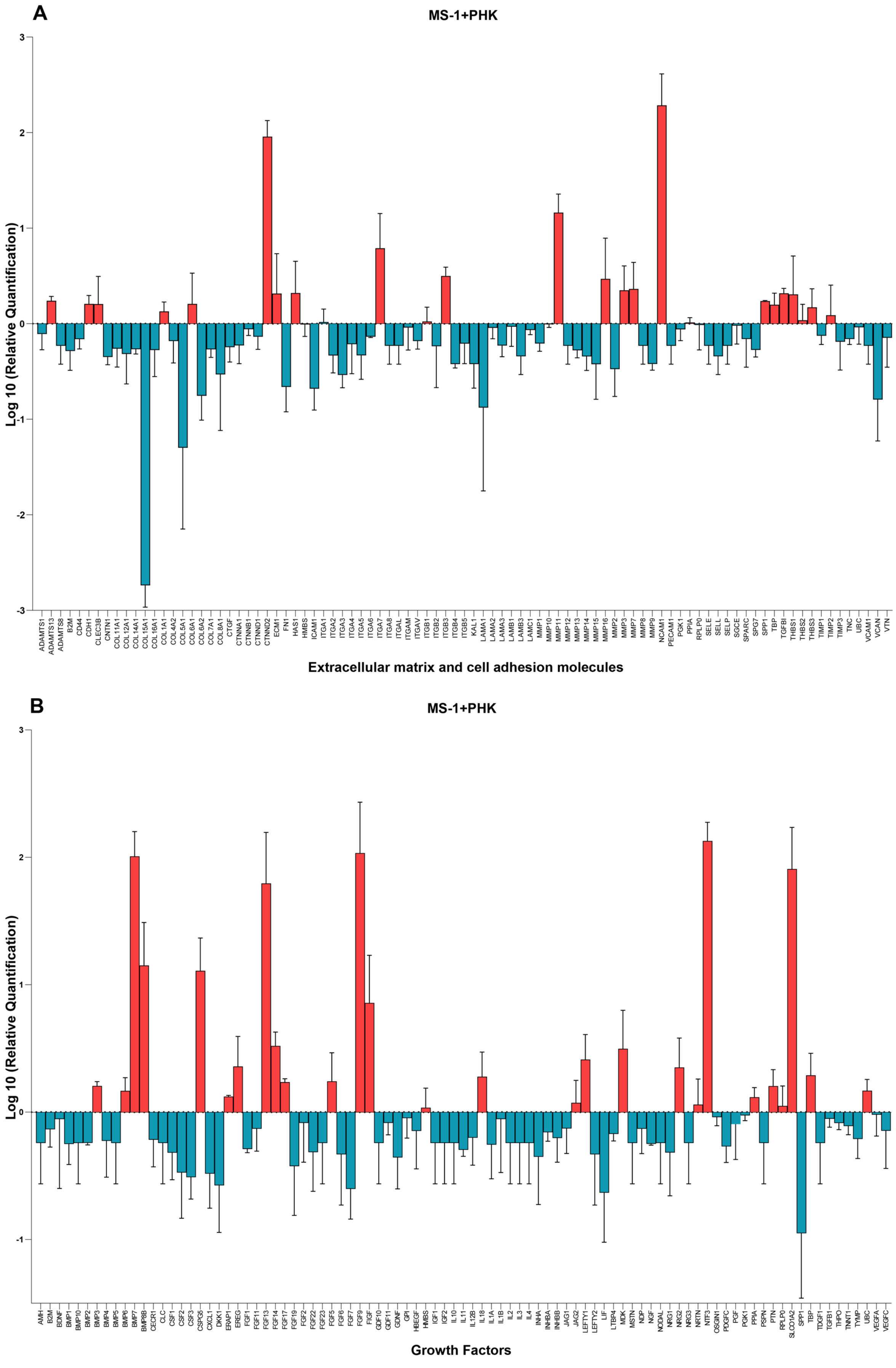

Figure S4. Gene expression profiles of co-cultures of MS-1 cells with primary human keratinocytes (PHKs). The plots depict the fold-change expression of (**A**) extracellular matrix and cell adhesion molecules and (**B**) growth factors relative to their expression in OERCs of PHKs. Data represent mean values ± SD of three independent experiments.

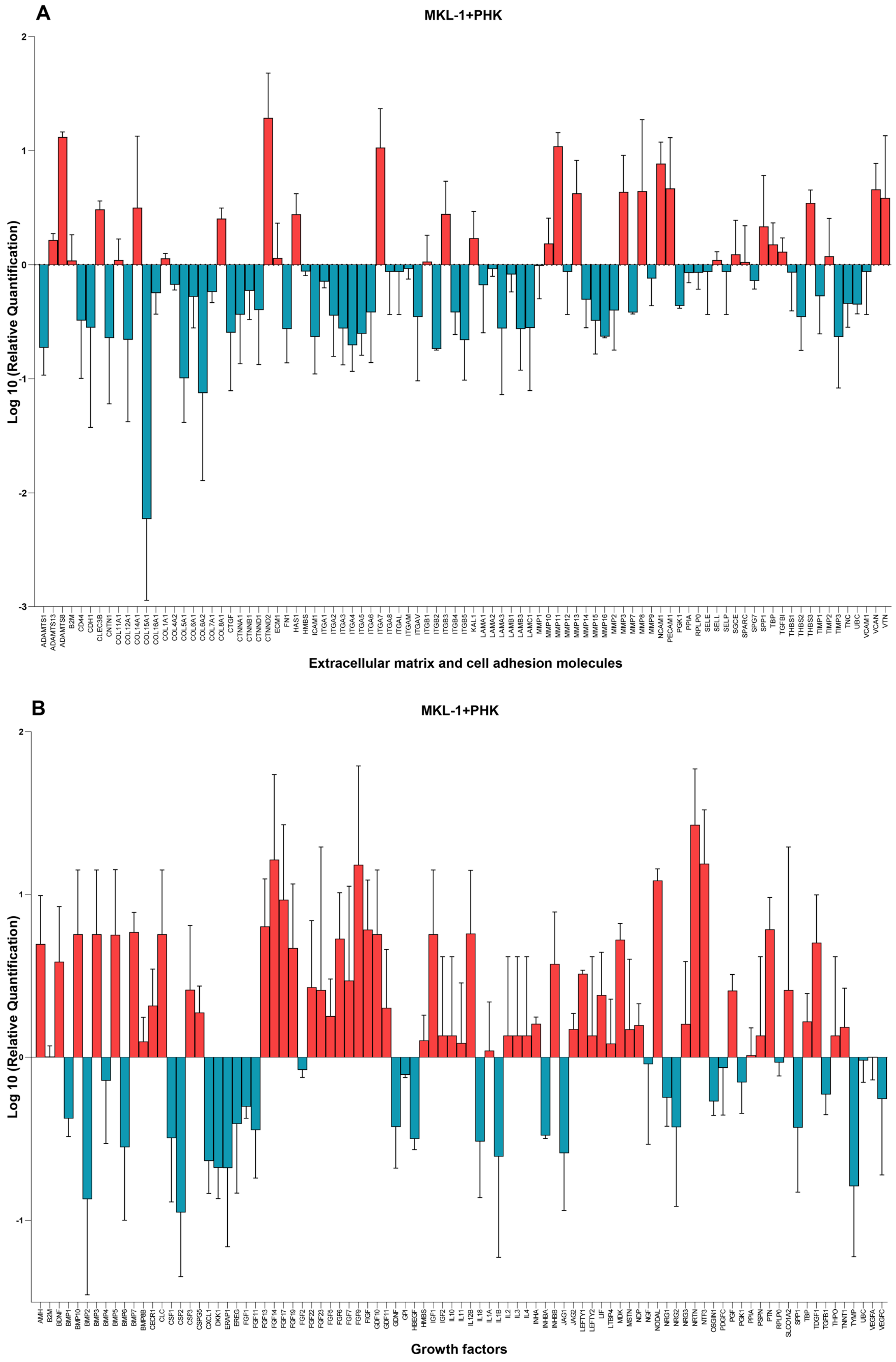

Figure S5. Gene expression profiles of co-cultures of MKL-1 cells with primary human keratinocytes (PHKs). The plots depict the fold-change expression of (**A**) extracellular matrix and cell adhesion molecules and (**B**) growth factors relative to their expression in OERCs of PHKs. Data represent mean values ± SD of three independent experiments.

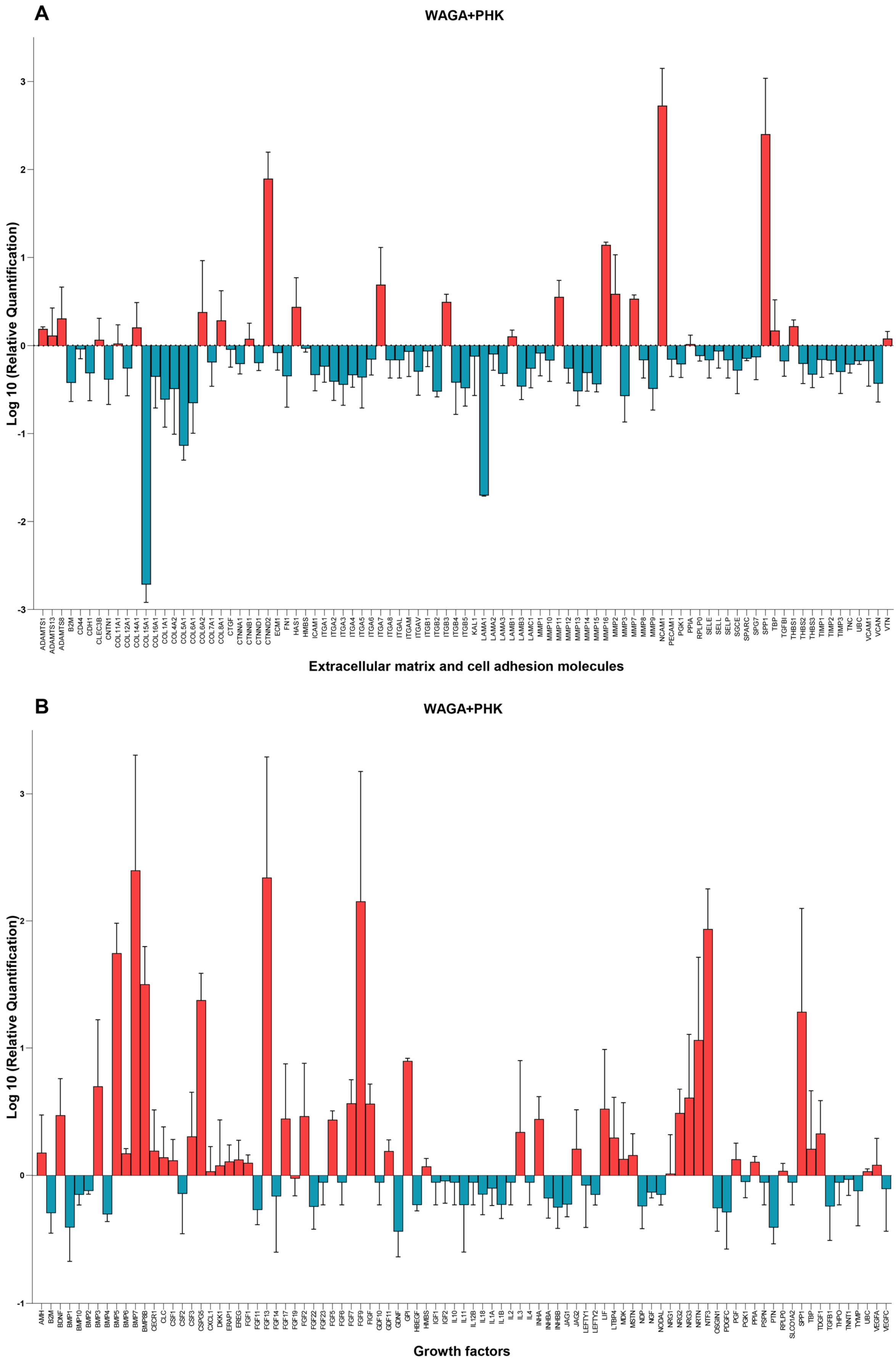

Figure S6. Gene expression profiles of co-cultures of WAGA cells with primary human keratinocytes (PHKs). The plots depict the fold-change expression of (**A**) extracellular matrix and cell adhesion molecules and (**B**) growth factors relative to their expression in OERCs of PHKs. Data represent mean values ± SD of three independent experiments.
